# *TP53* mutation variant allele frequency of ≥10% is associated with poor prognosis in therapy-related myeloid neoplasms

**DOI:** 10.1038/s41408-023-00821-x

**Published:** 2023-04-11

**Authors:** Mithun Vinod Shah, Elizabeth Ngoc Hoa Tran, Syed Shah, Rakchha Chhetri, Anmol Baranwal, Dariusz Ladon, Carl Shultz, Aref Al-Kali, Anna L. Brown, Dong Chen, Hamish S. Scott, Patricia Greipp, Daniel Thomas, Hassan B. Alkhateeb, Deepak Singhal, Naseema Gangat, Sharad Kumar, Mrinal M. Patnaik, Christopher N. Hahn, Chung Hoow Kok, Ayalew Tefferi, Devendra K. Hiwase

**Affiliations:** 1 Division of Hematology, Mayo Clinic, Rochester, MN USA; 2 Precision Medicine Theme, South Australian Health and Medical Research Institute (SAHMRI), Adelaide, SA Australia; 3 University of Adelaide, Adelaide, SA Australia; 4 Royal Adelaide Hospital, Central Adelaide Local Health Network, Adelaide, SA Australia; 5 Genetics and Molecular Pathology, SA Pathology, Adelaide, SA Australia; 6 Centre for Cancer Biology, University of South Australia and SA Pathology, Adelaide, SA Australia; 7 Division of Hematopathology, Department of Laboratory Medicine and Pathology, Mayo Clinic, Rochester, MN USA

**Keywords:** Medical research, Health care

## Abstract

Revised diagnostic criteria for myeloid neoplasms (MN) issued by the International Consensus Classification (ICC) and the World Health Organization (WHO) recommended major change pertaining to *TP53*-mutated (*TP53*^mut^) MN. However, these assertions have not been specifically examined in therapy-related myeloid neoplasm (t-MN), a subset enriched with *TP53*^mut^. We analyzed 488 t-MN patients for *TP53*^mut^. At least one *TP53*^mut^ with variant allele frequency (VAF) ≥ 2% with or without loss of *TP53* locus was noted in 182 (37.3%) patients and 88.2% of *TP53*^mut^ t-MN had a VAF ≥10%. *TP53*^mut^ t-MN with VAF ≥ 10% had a distinct clinical and biological profile compared to both *TP53*^mut^ VAF < 10% and wild-type *TP53* (*TP53*^wt^) cases. Notably, *TP53*^mut^ VAF ≥ 10% had a significantly shorter survival compared to *TP53*^wt^ (8.3 vs. 21.6 months; *P* < 0.001), while the survival of *TP53*^mut^ VAF < 10% was comparable to *TP53*^wt^. Within *TP53*^mut^ VAF ≥ 10% cohort, the inferior outcomes persisted irrespective of the single- or multi-hit status, co-mutation pattern, or treatments received. Finally, survival of *TP53*^mut^ patients was poor across all the blast categories and MDS patients with >10% blasts had inferior survival compared to <5%. In summary, *TP53*^mut^ VAF ≥10% signified a clinically and molecularly homogenous cohort regardless of the allelic status.

## Introduction

Tumor protein p53 (*TP53*) located on chromosome 17p13 is frequently mutated in cancer, including myeloid neoplasms (MN). Approximately 7–11% of *de novo* myelodysplastic syndromes (MDS) and 10–13% of acute myeloid leukemia (AML) patients harbor *TP53* mutations (*TP53*^mut^) [1–15]. *TP53*^mut^ MN are often associated with the features of genomic instability such as complex and monosomal karyotype (CK and MK, respectively) and are universally associated with adverse outcomes [1, 2, 4–11, 13, 15]. Therapy-related myeloid neoplasms (t-MN) are rare, but often fatal MN that develop following exposure to cytotoxic therapies [7, 12, 16] and are highly enriched in *TP53*^mut^, CK, and MK [17].

The recently published 5th edition of the World Health Organization classification of MN (WHO-5) [18], and the International Consensus Classification (ICC) [19] recommended major reorganization of the MN. Among the congruent changes, both WHO-5 and ICC recognize the poor prognostic impact of biallelic *TP53*^mut^ defined by the presence of ≥2 mutations or 1 mutation with the loss of residual wild-type *TP53* (*TP53*^wt^). Both classifications consider variant allele frequency (VAF) ≥ 50% as presumptive evidence of biallelic/multi-hit *TP53*^mut^, and biallelic *TP53*^mut^ MDS to be AML equivalent for therapeutic purposes. However, there are critical differences between the two classifications about allelic-status, VAF cut-off and blast categories [18, 19].

Another major change highlights the importance of genetic driver(s) and reduces the importance of the antecedent history and/or therapy. For example, ICC [19] removed t-MN as a distinct category and replaced it with a diagnostic qualifier, whereas WHO-5 [18] grouped t-MN with secondary MN as MN-post cytotoxic therapy (AML-pCT and MDS-pCT).

The underlying assumption of these changes is that *TP53*^mut^ MN are characterized by similar characteristics and outcomes. However, the studies driving these changes were highly enriched in *de novo* MN [2], excluded patients with <10% blasts [3] or >20% blasts [4], or only included MN with CK [14]. For example, in a cohort of predominantly of *de novo* MDS, single-hit *TP53*^mut^ had outcomes similar to *TP53*^wt^, whereas the association with CK, high risk of AML transformation, and poor survival were limited to multi-hit *TP53*^mut^ patients [2]. Meanwhile, *TP53*^mut^ AML and MDS with excess blasts (MDS-EB) had equally poor survival irrespective of single or multi-hit *TP53*^mut^ status [3]. Furthermore, in MDS and AML with CK, the single- or multi-hit *TP53*^mut^ was the only disease-related factor predicting survival [14]. We recently demonstrated that *TP53*^mut^ t-MN is associated with poor survival irrespective of single or multi-hit status [20], suggesting that the prognostic impact of allelic loss of *TP53*^mut^ MN is context dependent.

Hence, we performed a comprehensive analysis of a *TP53*^mut^ t-MN cohort to: (i) define the genomic landscape of *TP53*^mut^ t-MN; (ii) study the interaction of *TP53*^mut^ with BM blast % and structural chromosomal changes; (iii) study the impact of 17p loss in the absence of a concurrent *TP53*^mut^, and (iv) identify the optimal *TP53*^mut^ VAF threshold in t-MN.

## Methods

This retrospective multi-center study was conducted by Mayo Clinic, Rochester (USA) and the South Australia MDS Registry (SA-MDS, Australia) and includes all t-MN patients who had conventional G-banding chromosome analysis (CBA) and mutation testing using targeted sequencing of the most recurrently mutated genes in MN (please refer to supplementary section for details) [12, 21]. The respective databases captured patient-level information that included diagnostic characteristics, treatments including the use of allogeneic stem cell transplant, the response to therapies, and long-term follow-up.

Integrated genomic analysis that includes acquired copy-number abnormalities (CNA) analysis based on NGS data, SNP-array and FISH was performed in a subset of patients.

### Statistical methods

Comparisons were performed using Mann-Whitney U-test for non-normally distributed variables. Fisher’s exact test was used to determine associations between categorical variables. Overall survival (OS) was calculated from date of t-MN diagnosis to the last follow-up or the date of death. Post-transplant survival for patients who underwent allogeneic stem cell transplantation (SCT) was assessed from day of transplantation. Kaplan-Meier estimations were used with comparisons using log-rank tests. Cox regression multivariable analysis with backward selection was undertaken. *P* values < 0.05 were considered statistically significant. Further details of statistical analysis are provided in the Supplementary Methods section.

### Data Sharing Statement

Additional methods and data can be found in the Supplementary Methods section. For original data, please contact devendra.hiwase@sa.gov.au or Shah.Mithun@mayo.edu.

## Results

### Patient cohort

This international cohort of 488 t-MN patients included 318 with t-MDS (65.2%) and 170 with t-AML (34.8%). The median age at t-MN diagnosis was 68 (IQR 60, 74) years. The most common primary cancers were lymphoproliferative disorders (*n* = 142, 29.1%), plasma cell neoplasms (*n* = 64, 13.1%), breast cancer (*n* = 61, 12.5%), and prostate cancer (*n* = 39, 8%). Most common DNA-damaging therapies used for treating the primary disease were chemotherapy (*n* = 230, 47.1%), chemotherapy plus radiotherapy (*n* = 160, 32.8%), autologous SCT (*n* = 95, 19.5%), or radiation therapy alone (*n* = 79, 16.2%, Table S1).

The median latency from the time of primary disease to t-MN diagnosis, was 81 (IQR 40, 149) months. Following t-MN diagnosis, most patients were treated with disease-modifying therapies (DMT) including hypomethylating agents (HMA) (*n* = 160, 33.8%), intensive chemotherapy (*n* = 100, 20.5%), venetoclax-based therapies (*n* = 70, 14.3%), and 91 (18.6%) patients underwent allogeneic SCT (Table S1).

We first analyzed genome-wide allelic imbalances to include arm-level alterations detected by CBA. In agreement with prior studies [22, 23], 365 (76%) had at least one chromosomal aberration including CK (*n* = 190, 39.3%), MK (*n* = 183, 37.90%), deletion 7q or monosomy 7 (*n* = 148, 30.6%), deletion 5q or monosomy 5 (*n* = 108, 22.4%), and deletion 17p across *TP53* locus (referred to as 17p loss hereafter, *n* = 58, 12%) **(**Figure S1A**)**. We next analyzed the somatic mutation landscape of *TP53*^mut^ in t-MN.

### Genomic landscape of *TP53*^mut^ t-MN

**Fig. 1:**
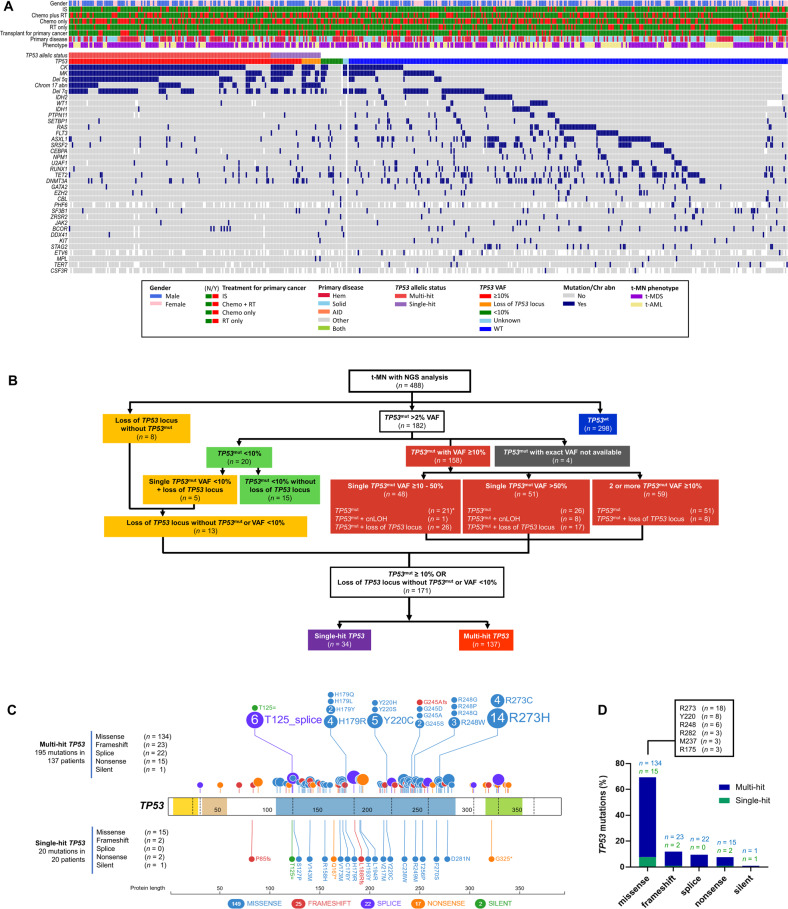
Landscape of *TP53* mutation (*TP53*^mut^) in therapy-related myeloid neoplasms (t-MN). **A** Oncoplot showing cytogenetic and mutational landscape of *TP53*^mut^ and wild-type *TP53* (*TP53*^wt^) t-MN. Patient-related factors (t-MN phenotype, abnormal karyotype, primary disease, treatment status, and gender) are shown in the upper panel, and the distribution of somatic gene mutations (including *TP53* mutation status) in the lower panel. Each column represents an individual patient, and the presence of the aberration or mutation is colored as indicated above; **B** Consort diagram of the mutant *TP53* status of 488 t-MN patients analyzed by integrated analysis employing next gene sequencing (NGS), conventional cytogenetics, FISH, SNP-array and CNA analysis. *Of the 48 patients with single *TP53*^mut^ VAF 10–50% LOH information was available in 33 patients. Importantly, 80% of the remaining *TP53*^mut^ patients (*n* = 15) without LOH information has complex karyotype and are considered equivalent to multi-hit by ICC; **C** Distribution of *TP53*^mut^ along the gene. Mutations from single-hit patients are shown at the bottom and those from multi-hit patients are shown at the top. Missense mutations are shown as blue circles, truncated mutations corresponding to nonsense mutations as orange circles, frameshift deletions or insertions as red circles, and splice site variants are shown as purple circles. Other types of mutations are shown as green circles. Functional protein domains are indicated in yellow (transactivation motif), brown (transactivation domain 2), blue (DNA binding domain), and green (tetramerization motif); **D** Summary of *TP53*^mut^ separated by mutation type and frequency of the mutations.

The next generation sequencing (NGS) identified 253 putative oncogenic mutations in *TP53* at VAF ≥ 2% in 182 (37.29%) patients (Fig. 1A, B). A diverse spectrum of *TP53*^mut^ including highly frequent missense mutations followed by frameshift insertion-deletions, splice-site, and nonsense mutations was observed (Fig. 1C, D and Figure S1B, C). Nearly all missense mutations occurred in the *TP53* DNA-binding domain (Fig. 1C and Figure S1B).

**Fig. 2:**
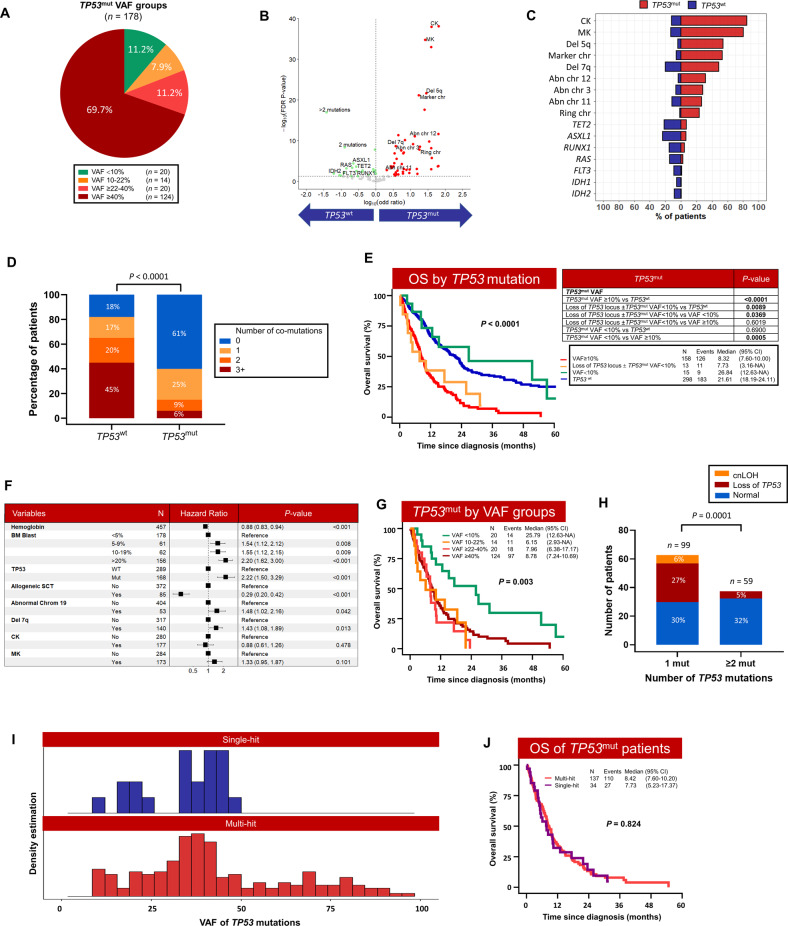
*TP53*^mut^ drive genomic instability and was associated with poor overall survival in therapy-related myeloid neoplasms (t-MN). **A** Distribution of cases according to *TP53*^mut^ VAF; **B** Volcano plot comparing cytogenetic aberration and somatic mutations in *TP53*^mut^ and *TP53*^wt^ t-MN. Chromosomal aberrancies highly prevalent in *TP53*^mut^ (red) and somatic mutations enriched in *TP53*^wt^ cohort (green). Genomic changes that are not differentially expressed between the two groups are shown in gray color; **C** Frequency of cytogenetic aberrations or driver oncogenic gene mutations in *TP53*^wt^ and *TP53*^mut^ t-MN; **D** Number of co-mutations in *TP53*^wt^ and *TP53*^mut^ t-MN; **E** Overall survival (OS) of *TP53*^mut^ with VAF ≥10% or loss of *TP53* locus was significantly poor compared to wild-type *TP53* (*TP53*^wt^) and *TP53*^mut^ with VAF < 10% t-MN; **F** Multivariate Cox-regression analysis of factors predicting overall survival in t-MN; **G** OS of *TP53*^mut^ t-MN according to VAF cut-offs; **H** Frequency of loss of heterozygosity (LOH) and copy neutral LOH (cnLOH) according to number of *TP53*^mut^; **I** Density estimation of VAF of single-hit and multi-hit *TP53*^mut^; **J** OS is equally poor in single- and multi-hit in t-MN.

In the majority of the *TP53*^*mut*^ t-MN with available information (*n* = 178; 97.8%), *TP53*^mut^ was a dominant driver clone with VAF ≥ 40% (*n* = 124; 69.7%), while 20 (11.2%), 14 (7.8%) and 20 (11.2%) patients had VAF ≥ 22–40%, 10–22% and <10%, respectively (Fig. 2A).

Next, we determined the *TP53*^mut^ VAF threshold associated with poor prognosis in t-MN. The receiver operating characteristics analysis identified 8% as the optimal VAF threshold (Figure S2A–C). As only 5 (2.3%) patients had VAF 8–10%, we adapted the ICC VAF threshold of 10% for further analyses (Figure S2C, D).

### *TP53*^mut^ VAF ≥ 10% is associated with distinct presentation, features of genomic instability, and outcomes in t-MN

The majority of *TP53*^*mut*^ t-MN had VAF ≥10% (*n* = 158; 88.8%) (Fig. 1B). The loss of 17p across *TP53* locus (LOH) or copy neutral LOH (cnLOH) was detected in 73 (38.4%) patients including *TP53*^mut^ VAF ≥10% (*n* = 60), VAF < 10% (*n* = 5), and in the absence of *TP53*^mut^ (*n* = 8). Collectively, 171 (35%) had *TP53*^mut^ VAF ≥ 10% or LOH/cnLOH across the *TP53* locus.

We next compared cytogenetics abnormalities and somatic mutations in 30 genes analyzed in both (South Australian and Mayo) cohorts. Genomic instability was highly evident in *TP53*^mut^ with VAF ≥ 10% and/or loss of *TP53* locus compared to *TP53*^wt^ t-MN. CK, MK, chromosome 5 aberrancies, and marker chromosomes were enriched in *TP53*^mut^ t-MN (Fig. 2B, C, Table S2). In contrast, recurrent oncogenic mutations such as *ASXL1*, *DNMT3A*, *FLT3-ITD*, *IDH1*, *IDH2*, *NPM1*, *PTPN11*, *RAS*, *RUNX1* and *TET2* were less frequent in *TP53*^mut^ t-MN (Figs. 1A, 2B, C, Table S2). Moreover, total number of co-mutations were significantly less in *TP53*^mut^ cases compared to *TP53*^wt^ (*P* < 0.001) (Table S2 and Fig. 2D). Enrichment of chromosomal aberrancies and lower frequency of somatic mutation was observed in *TP53*^mut^ complex karyotype MDS [4] and multi-hit *TP53*^mut^ compared to single-hit *TP53*^mut^ and *TP53*^wt^ *de novo* MDS [2]. *TP53*^mut^ not only influenced the genomic instability but also dictated the clinical presentation. *TP53*^mut^ t-MN had more severe anemia (*P* < 0.001), leukopenia (*P* < 0.001), and thrombocytopenia (*P* < 0.001) (Table S2).

Importantly, patients with *TP53*^mut^ VAF ≥ 10% with or without loss of *TP53* locus had significantly shorter survival compared to *TP53*^wt^ (8.3 *vs*. 21.6 months; *P* < 0.001) (Fig. 2E). The three-year overall survival was 7% in patients with *TP53*^mut^ compared to 34% in *TP53*^wt^ patients (*P* < 0.0001). Poor survival of *TP53*^mut^ was observed in t-MDS (9.9 vs. 24.1 months; *P* < 0.001) and t-AML (3.6 vs. 13.2; *P* < 0.001) (Figure S3A, B). The inferior outcomes of *TP53*^mut^ persisted across all the t-MN treatment types, including supportive care (3.9 vs. 19.1; *P* < 0.0001), intensive chemotherapy (7.3 vs. 23.1; *P* < 0.0001), hypomethylating agents (10.9 vs. 20.5; *P* = 0.001), venetoclax-based combination therapies (8.1 *vs*. 23.3; *P* = 0.01) and allogenic SCT (20.6 *vs*. not reached; *P* = 0.01) (Figure S4A–F). Univariate Cox-regression analysis suggested that *TP53*^mut^, specific chromosomal abnormalities, complex karyotype, bone marrow blasts, age and allogeneic SCT predicted OS of t-MN (Table S3). Furthermore, multivariable multivariate Cox regression analyses validated inferior survival of *TP53*^mut^ (HR 2.18, 95% CI 1.47–3.25; *P* < 0.001), independent of BM blast percentage (*P* = 0.003), chromosome 19 (*P* < 0.001) and allogeneic SCT (*P* < 0.001) (Fig. 2F). Poor prognosis with associated with *TP53*^mut^ and higher blast counts is known in AML and MDS, however prognostic impact of abnormalities in chromosome 19 are not well known. In contrast to *de novo* AML [24] and MDS [25], chromosome 19 was associated with poor survival in t-MN.

In contrast to *TP53*^mut^ with VAF ≥ 10%, majority of the clinical, cytogenetic, and mutation profile were similar between *TP53*^mut^ VAF < 10% and *TP53*^wt^ t-MN, except lower BM blasts and higher frequency of del 5q, CK, and ring chromosome. While somatic mutations were more prevalent in *TP53*^wt^ t-MN (Table S4). Importantly, OS was not significantly different between the two groups (26.8 *vs*. 21.6 months, *P* = 0.69; Fig. 2E). In contrast, chromosomal aberrancies and OS were significantly different in *TP53*^mut^ patients when categorized according to VAF < 10% vs. ≥10% (Fig. 2E and Table S5). Surprisingly, OS was equally poor in *TP53*^mut^ when VAF 10–22%, 22–40%, and ≥40% (6.15 vs. 7.96 vs. 8.78 months, Fig. 2G) cut-offs were used.

### Majority of the *TP53*^mut^ t-MN harbor biallelic loss of *TP53*

Among the 158 patients with *TP53*^mut^ VAF ≥ 10%, 70% had *TP53*^mut^ plus LOH/cnLOH of *TP53* locus (*n* = 52; 32.9%) or ≥2 *TP53*^mut^ (*n* = 59; 37.3%), while 29.1% (*n* = 47) had single *TP53*^mut^ (Fig. 2H). Of the 47 patients with single *TP53*^mut^, 26 (55.3%) and 21 (44.7%) patients had VAF > 50% and 10–50%, respectively (Fig. 1B). Additional 13 patients had loss of the *TP53* locus without evidence of *TP53*^mut^ (*n* = 8) or with *TP53*^mut^ VAF < 10% (*n* = 5) (Fig. 1B). Frequency of LOH/cnLOH was significantly higher in cases with single *TP53*^mut^ compared to cases with ≥2 *TP53*^mut^ (33% *vs*. 5%, *P* = 0.0001; Fig. 2H).

Next, we compared the clinical features, profiles of genome stability and patterns of co-mutation for each *TP53* allelic state. Integrated cytogenetic, copy number and somatic mutation analysis classified *TP53*^mut^ as multi-hit if there is: (1) presence of ≥2 distinct *TP53*^mut^, each with VAF ≥ 10%, or (2) a single *TP53*^mut^ associated with either: (i) cytogenetic deletion of 17p13 involving the *TP53* locus; (ii) a VAF of >50%; or (iii) copy-neutral loss of heterozygosity (cnLOH) at the *TP53* locus. Single *TP53*^mut^ with VAF 10%-50% or loss of 17p13 involving *TP53* locus without *TP53*^mut^ were defined as single-hit. In total, 34 (19.9%) of the 171 patients with *TP53*^mut^ and/or loss of *TP53* locus were considered single-hit and 137 (80.1%) were multi-hit (Fig. 1B). In single-hit *TP53*^mut^ cases, the median VAF was significantly lower compared to multi-hit *TP53*^mut^ (34% *vs*. 38.2%, *P* = 0.006) (Table S6 and Fig. 2I). Overall, the spectrum of *TP53*^mut^ was shared among single- and multi-hit states (Fig. 1C).

Unlike *de novo* MDS [2], there was no significant difference in CK, MK, CK plus MK, chromosome 5 aberrancy, or co-mutations between single- and multi-hit *TP53*^mut^ (Table S6). We did not observe significant differences in clinical features, age, latency, blood counts, BM blast percentage and cytogenetics when stratified by the allelic status except that the multi-hit *TP53*^mut^ were enriched for marker chromosome. In contrast to previous publications [2, 14], the distribution of single- and multi-hit *TP53*^mut^ was not different across the t-MN phenotype and the BM blast categories (Figure S5A, B).

Consistent with our previous observation [20], the OS was not significantly different between the single- and multi-hit *TP53*^mut^ t-MN (Fig. 2J). Similarly, there was no survival difference between single- and multi-hit *TP53*^mut^ when stratified by t-MDS *vs*. t-AML, according to the blast cut-off proposed by ICC (Figure S6A–D), or the type of treatment received (Figure S7A–C). Finally, there was no difference in the incidence of progression to AML in single versus multi-hit *TP53*^mut^ t-MDS (Figure S7D).

### *TP53*^*mut*^ burden increases with number of chromosomal aberrancies

**Fig. 3:**
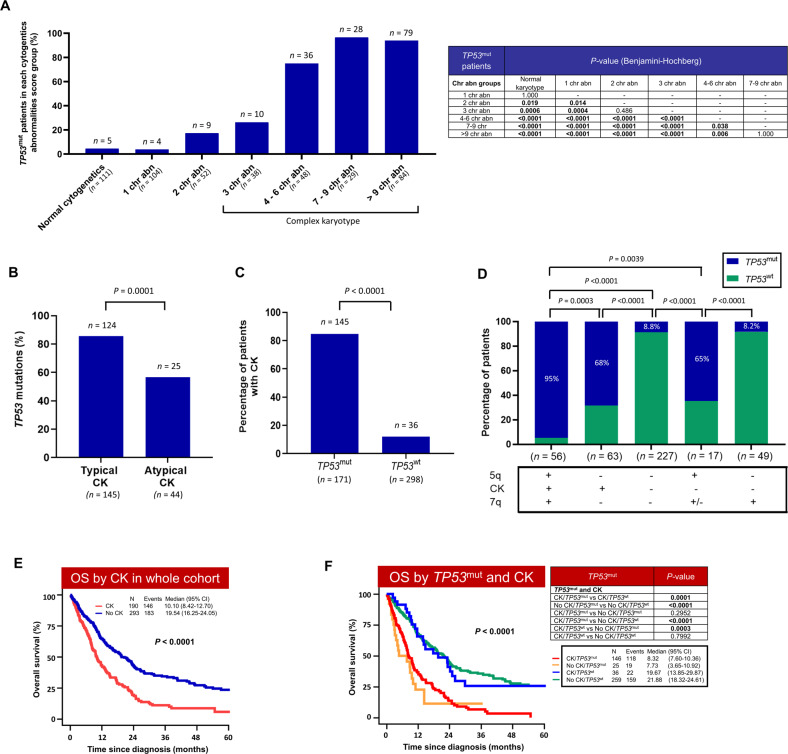
*TP53*^mut^ was enriched in complex karyotype (CK) and is associated with significantly poor outcome of CK t-MN. **A** Frequency of *TP53*^mut^ according to number of structural cytogenetic abnormalities; **B** High frequency of *TP53*^mut^ in typical-CK compared to atypical-CK t-MN; **C** *TP53*^mut^ cases were enriched for CK; **D** In the absence of CK, *TP53*^mut^ were prevalent in cases with 5q loss compared to 7q loss (+, present; -; absent; +/-, present or absent). Loss of 5q was defined as monosomy 5 or del 5q, while loss of 7q was defined as monosomy 7 or del 7q; **E** CK is associated with significantly poor survival in t-MN; **F** *TP53*^mut^ status further stratify CK with very poor outcome.

The proportion of *TP53*^mut^ increased from 4.5% in normal karyotype cases to 17.3% in cases with two chromosomal aberrancies (*P* = 0.019; Fig. 3A) and 76.8% in cases with CK (*P* < 0.0001). Even within the CK group, enrichment of *TP53*^mut^ was observed with the increasing number of cytogenetic abnormalities: from 26.3% in cases with three chromosomal abnormalities to 75%, 96.6%, and 94% in cases with 4–6, 7–9, and >9 chromosomal aberrancies (Fig. 3A and Fig. S8). More than 80% of *TP53*^mut^ were segregated in t-MN with >4 cytogenetic abnormalities (Figure S8). Furthermore, *TP53*^mut^ were significantly high in typical- compared to atypical-CK (Fig. 3B). Typical CK is defined as CK with ≥3 abnormalities that include 5q, 7q, and/or 17p loss and atypical-CK as CK with ≥3 abnormalities without these specific abnormalities [26]. Conversely, *TP53*^mut^ were enriched for CK compared to *TP53*^wt^ (84.8% *vs*. 12.0%, *P* < 0.0001; Fig. 3C).

The enrichment of *TP53*^mut^ was also noted in cases with del 5q without CK. However, such enrichment of *TP53*^mut^ was not observed in del 7q without CK (Fig. 3D). Together these findings indicate that *TP53*^mut^ burden increases not only with number but with type of chromosomal aberrancies.

Complex karyotype is associated with poor survival (10.1 *vs*. 19.5 months; *P* < 0.001) (Fig. 3E). We assessed if *TP53*^mut^ can further stratify by CK t-MN. Even within CK, *TP53*^mut^ is associated with a higher structural genomic instability. In particular, MK, marker chromosomes, ring chromosome, 5q del/monosomy 5, chromosome 12, and 18 abnormalities were highly prevalent in *TP53*^mut^-CK compared to *TP53*^wt^-CK (Table S7). While number of somatic mutations, including *RAS*, *ASXL1*, and *RUNX1* were enriched in *TP53*^wt^-CK (Table S7). Importantly, *TP53*^mut^ further stratified outcomes for CK, with inferior survival of *TP53*^mut^-CK compared to the *TP53*^wt^-CK (8.3 *vs*. 19.7 months; *P* < 0.001, Fig. 3F). Conversely, CK-status did not influence the poor outcome of *TP53*^mut^ t-MN (8.3 vs. 7.7 months, *P* = 0.29; Fig. 3F). Furthermore, OS of *TP53*^wt^ CK was not significantly different than non-CK *TP53*^wt^. Together these findings suggest that poor prognosis of CK is driven by its association with prognostically adverse *TP53*^mut^ (Fig. 3F). Similar association was previously reported in CK-MDS [4].

### *TP53*^mut^ t-MDS can be stratified according to ICC BM blast categories

**Fig. 4:**
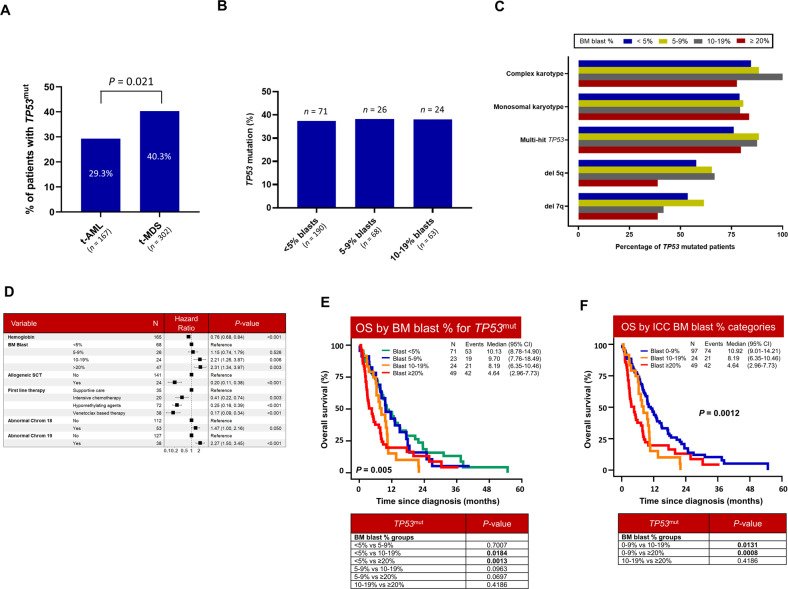
Interaction between *TP53*^mut^ and bone marrow (BM) blast percentage. **A** Distribution of t-AML and t-MDS according to *TP53*^mut^ status; **B** *TP53*^mut^ frequency in t-MDS was similar across blast categories at diagnosis; **C** Chromosomal aberrancies and *TP53*^mut^ allelic status across BM blast %; **D** Multivariate Cox-regression analysis showing BM blast >10%, chromosomal 18/19 abnormalities and disease modifying therapies were independent predictors of *TP53*^mut^ OS; **E** In *TP53*^mut^ t-MN, BM blast 10–19% and ≥20% were associated with poor OS compared to BM blast <5%; (F) OS of *TP53*^mut^ t-MN according to ICC blast categories (0–9% vs. 10–19% *vs*. ≥20%).

Next, we evaluated the frequency of *TP53*^mut^ according to the disease phenotype. *TP53*^mut^ burden was significantly higher in t-MDS compared to t-AML (40.3% vs. 29.3%, *P* = 0.021; Fig. 4A). However, the frequency of *TP53*^mut^ in t-MDS was similar across the BM blast categories: 37.3 *vs*. 38.2 *vs*. 38.1% in <5%, 5–9% and 10–19% blasts, respectively (Fig. 4B and Table S8). There was no significant difference in age at t-MN diagnosis, latency, the type of the primary disease, or the degree of cytopenia at t-MN diagnosis across the blast % categories (Table S8). Moreover, genomic instability as evidenced by CK, MK, and number of cytogenetic abnormalities were also similar across all four categories (Table S8, Fig. 4C). Finally, the proportion of patients with single- *vs*. multi-hit, *TP53* VAF, and co-mutations were comparable across the four blast categories (Table S8).

Due to the molecular homogeneity of *TP53*^mut^ across the blast categories, we investigated prognostic impact of BM blast %, variants in cancer-related genes, cytogenetics, and the severity of cytopenia in *TP53*^mut^ t-MN. BM blasts 10–19%, ≥20%, hemoglobin, chromosome 19 abnormalities, and the first line therapies including allogeneic SCT were independent predictors of outcome in *TP53*^mut^ t-MN (Fig. 4D**;** Table S9). Importantly, OS of *TP53*^mut^ patients was poor across the blast categories with statistically significant difference between the BM blasts <5% *vs*. 10–19% and <5% *vs*. ≥20% categories (Fig. 4E). The OS of *TP53*^wt^ t-MN significantly worsened with increasing blast % (*P* = 0.001) (Figure S9).

Recently, ICC proposed three categories of *TP53*^mut^ MN according to BM blast % [19]. OS was significantly different in t-MDS with blast 0–9% *vs*. 10–19% blasts (10.9 vs. 8.2 months; *P* = 0.01). Similarly, OS was significantly different in *TP53*^mut^ t-MN with BM blast 0–9% vs. ≥20% (10.9 *vs*. 4.6 months, *P* = 0.0008) (Fig. 4F). Together these findings suggest that *TP53*^mut^ t-MN with >10% is uniformly associated with poor survival.

## Discussion

The results above collectively demonstrate that the classification of t-MN based on *TP53* status is clinically and biological relevant. This conclusion is supported by our findings that (1) *TP53*^mut^ t-MN with ≥10% is uniformly associated with poor survival; (2) the presence of *TP53*^mut^ was an independent risk factor for poor survival and was associated with inferior outcome, even within the traditionally known high-risk subsets such as CK; (3) the inferior outcomes persisted irrespective of the single- or multi-hit status, the co-mutation pattern, or the treatments received; (4) poor survival of single and muti-hit *TP53*^mut^ was observed across the blast categories of t-MN.

*TP53*^mut^ influenced the clinical presentation and survival. Although severe cytopenia is reported across multiple studies [2, 4, 14], the association with BM blast percentage is debated [2, 4, 10, 27]. *TP53*^mut^ *de novo* MDS present with high BM blast [4, 27], especially those with the multi-hit alterations [2]. However, MDS with *TP53*^mut^/CK present with low BM blast burden [14]. We observed that *TP53*^mut^ patients were more likely present as t-MDS. Furthermore, within the t-MDS cohort, BM blast burden was lower in *TP53*^mut^ cases. In contrast to *de novo* MDS [2], frequency of multi-hit *TP53*^mut^ remained similar across the BM blast categories. Secondly, poor prognosis of multi-hit *TP53*^mut^ MDS/AML was reported irrespective of the BM blast and therapy-relatedness [14]. In our cohort, OS of *TP53*^mut^ t-MN was poor across all the blast categories irrespective single- or multi-hit status and type of disease modifying therapies. However, OS of *TP53*^mut^ MDS BM blasts >10% and AML was significantly inferior compared to *TP53*^mut^ BM blasts <10%. Thus, the interaction between BM blast and *TP53*^mut^ appears to be disease ontogeny specific. Overall, our findings support the ICC stratification of *TP53*^mut^ MN using three blast cut-offs.

Though poor survival of *TP53*^mut^ is well known, the prognostic implication *TP53*^mut^ VAF remains an active area of research. Multiple studies reported VAF > 40% is associated with poor survival in high-risk MDS [4, 10, 28] while one study suggested that poor survival is a direct function of increasing VAF as a continuous variable [7]. In contrast to these findings, other studies showed an inferior OS irrespective of *TP53*^mut^ VAF [3, 14, 29, 30]. Furthermore, prognostic implication of *TP53*^mut^ VAF also depend upon *TP53*^mut^ allelic status. Single-hit *TP53*^mut^ MDS with VAF > 22% had poor survival and the favorable survival comparable to the *TP53*^wt^ was restricted to the single-hit cases with VAF ≤ 22%. Conversely multi-hit patients had poor outcome across the range of *TP53* VAF [2]. In t-MN, OS was significantly poor in *TP53*^mut^ with VAF ≥ 10% compared to VAF < 10%. There was no survival difference in cases with VAF 10–22% *vs*. >22–40% *vs*. >40%. Together these findings suggest prognostic implication of *TP53*^mut^ VAF is context dependent and varies significantly between *de novo* and therapy-related MN.

As expected, *TP53*^mut^ and CK/MK were highly enriched in t-MN compared to *de novo* MDS [2] and AML [3, 5, 6, 31]. Within t-MN, CK was more frequent with *TP53*^mut^ than *TP53*^wt^. Conversely, increasing genomic instability was associated with enrichment of *TP53*^mut^: 75% and ~90% of patients with ≥4–6 and ≥7 chromosomal abnormalities harbored *TP53*^mut^, respectively, compared to only 26% of patients with 3 chromosomal abnormalities. In addition to number, type of chromosomal abnormalities also influences enrichment of *TP53*^mut^. Critical understanding of the relationship between *TP53*^mut^ and chromosomal aberrancies can be harnessed for prioritization of *TP53*^mut^ testing in limited resources, and screening/counselling appropriate patients for clinical trials of novel therapies as waiting time for mutation results can be up to 3–4 weeks. Importantly, within CK t-MN, patients with co-existent *TP53*^mut^ had evidence of profound genomic complexities and structural aberrancies [14, 32], and had poor outcomes compared to CK-*TP53*^wt^ in our t-MN cohort and other MN [10], emphasizing the importance of assessing *TP53*^mut^ alongside complex karyotype for an accurate risk estimation. Conversely, poor prognosis of biallelic *TP53* loss compared to single allelic loss was evident only in non-CK, while presence of CK was invariably associated with poor survival irrespective of *TP53*^mut^ allelic status [33].

In contrast to the findings in predominantly *de novo* MDS, we did not observe a difference in the frequency of structural chromosomal aberrancies including CK, MK, or co-mutation pattern between single- and multi-hit *TP53*^mut^ in t-MDS and as such in the whole t-MN cohort. Moreover, there was no difference in the transformation rate to AML and the OS between the multi- and the single-hit *TP53*^mut^ t-MDS. These findings have significant impact on the classification and management of t-MN patients especially considering the other recent changes in the WHO classification. The WHO has grouped t-MN with secondary MN and renamed it as “myeloid neoplasm post cytotoxic therapy”, with the assertion that a majority of MDS and AML occurring post-cytotoxic therapy have *TP53*^mut^ and that only multi-hit *TP53*^mut^ had a poorer outcome compared to single-hit [2, 18], thus undermining the poor prognosis of single-hit *TP53*^mut^ t-MN. Exclusion of single-hit *TP53*^mut^ t-MDS from the *TP53* mutated MDS have huge impact on management such as consideration for allogeneic stem cell transplantation [34], and exclusion from clinical trials targeted toward *TP53*^*mut*^ MDS. For example, allogeneic SCT may not be offered to fit single-hit *TP53*^mut^ t-MDS with BM blast 5–9% (according to ICC) and <20% (according to WHO) as they are considered to have OS similar to *TP53*^wt^ MDS. Similarly, these patients would be excluded from enrolment in clinical trials.

The apparent discrepancies in the conclusions among prior studies can be on the account of the characteristics of the study cohort, technical aspects, as well as true biological differences. For example, t-MN constituted a small subset of the patients in some large studies [2, 4]. Others excluded patients with low blasts [3], or only included patients with CK [4, 14]. Secondly, variable VAF thresholds have been used: 1% [3], 2% [2, 14], or 10% in the ICC guidelines. Thirdly, and likely the most significant difference is the criteria used to designate single *vs*. multi hit status. For example, in the absence of a detailed analysis of the *TP53* locus (using CBA, FISH, or chromosomal microarray), both WHO and ICC consider >50% VAF as presumptive evidence multi-hit, though the evidence suggests that VAF estimation is a poor surrogate for the allelic status [2]. In the absence of LOH information, the presence of a single *TP53*^mut^ in the context of CK is considered equivalent to a multi-hit *TP53*^mut^ by ICC, but not in WHO-5. Key studies driving recent classifications variably used CBA, FISH, NGS, and SNP array to determine the hit status [2, 3]. In contrast, a recent study used WGS to confer hit status [35]. Therefore, what LOH assessment is considered minimal or optimal is unclear at this time. In summary, there is an urgent need to define a uniform diagnostic genetic tools and criteria for determining allelic status of *TP53*^mut^ and VAF in all types of MN including t-MN, secondary and *de novo* MN.

Finally, the universally poor outcome of *TP53*^mut^ t-MN highlights the great unmet need for these patients and the necessity for novel therapies. Unfortunately, none of the routinely offered leukemia-directed therapies were able to overcome the impact of *TP53*^mut^. *TP53*^mut^ are noted to be present long before the eventual development of t-MN—sometimes even before the original exposure to DNA-damaging therapies. Recent evidence suggests a deterministic order of genetic and genomic changes following *TP53* mutation/loss [36, 37]. A comprehensive characterization of the genomic changes, and its correlation with the resultant morphological changes, may help identify patients at the risk of imminent leukemic transformation and devise effective preventive strategies. Hence, the identification of individuals at high-risk of developing t-MN and employment of preventative approaches may improvement outcomes for this difficult-to-treat group of patients [16, 38–40].

## Supplementary information

Supplementary materials
