# Supplementary material for: TP53 mutation variant allele frequency of ≥10% is associated with poor prognosis in therapy-related myeloid neoplasms
Source: Blood Cancer J. 2023 Apr 11;13(1):51. doi: 10.1038/s41408-023-00821-x (PMC10090194; doi:10.1038/s41408-023-00821-x)
Supplement: Supplementary file 1 — Supplementary materials [file 41408_2023_821_MOESM1_ESM.docx]

**Supporting Information for:**

***TP53* mutation variant allele frequency of ≥10% is associated with poor prognosis in therapy-related myeloid neoplasms**

**This pdf file contains:**

Methods

Tables S1 – S9

Figure S1 – S9

References

**SUPPLEMENTARY METHODS, TABLES & FIGURES**

**Methods**

**Patient samples.** Collaboration between Mayo clinic (Rochester, USA) and South Australian MDS/AML registry (Australia) led to this International Therapy-related Myeloid Neoplasm (t-MN) Registry. Under supervision of expert Hematologists, dedicated team members from both centers curated electronic and paper records of all t-MN patients diagnosed according to the WHO 2016 classification [1]. Patients (*n* = 488) with well curated clinical, demographic, blood counts, bone marrow reports, conventional G-banding cytogenetic and next generation sequencing of recurrently mutated genes in myeloid malignancies were included in this study. The source for genomic DNA was bone marrow mononuclear cells. Data was obtained with informed consent or appropriate consent waiver in accordance with the Declaration of Helsinki and appropriate Ethics Committee approval.

**Clinical data.** All data shared for this study were assigned unique patient identifiers and anonymized data including clinical variables such as (i) details of the primary disease treated with cytotoxic therapies, age at primary cancer/AID diagnosis, interval between primary cancer/AID and t-MN diagnosis (latency period); (ii) blood counts, bone marrow blasts, conventional G-banding karyotypes, pathogenic somatic mutations and WHO t-MN subtype (t-MDS, t-AML); (iii) treatment for t-MN and responses; (iv) clinical outcomes including the time of death from any cause or last follow-up from sample collection, and the time of AML transformation.

**Cytogenetics and other analyses.** Conventional karyotype analysis was carried out as described previously [2]. The cytogenetic data was manually curated by DH, RC, ET, DL and MVS as per the International Working Group for MDS Molecular Prognostic Committee [3,4].

Complex karyotype (CK) was defined as previously described [2]. Although any chromosome can participate in various structural and numerical abnormalities constituting CK, the involvement of particular chromosomes or chromosome arms in these aberrations is non-random. The most common aberrancies are chromosome 5 (5q), followed by 7q and 17p [5]. Deletions of 5q, 7q, and 17p often occur together. Hence CK were further categorized into typical and atypical categories. Typical CK was defined as CK with ≥3 abnormalities that include 5q, 7q, and/or 17p loss, and the atypical-CK as CK with ≥3 abnormalities other than these aberrancies [5,6]. Monosomal karyotype (MK) was defined by the presence of 2 or more monosomies (excluding loss of X or Y), or 1 monosomy plus at least 1 structural chromosomal aberration (excluding AML-associated recurrent cytogenetic abnormality) [7].

**Fluorescence in situ hybridization (FISH).** FISH analysis was performed to verify loss of *TP53* locus in cases with 17p deletions (*n* = 24) as described previously [2]. Cytogenetic and FISH findings were reported in accordance with the 2017 International System for Human Cytogenetic Nomenclature [8].

**SNP array***.* Microarray-based genomic profiling was performed on BM-MNC using Affymetrix CytoScan HD platform and Illumina CytoSNP-850K BeadArray v1.2. These arrays comprise approximately 750,000 and 850,000 SNP markers retrospectively across the whole genome. Following hybridization of PCR-amplified, enzymatically fragmented and labelled DNA to the complementary oligomers, the Cytoscan HD chip was imaged, and the resulting files were analysed using Chromosome Analysis Suite software (ChAS) and BlueFuse Multiv4.5 with genome build GRCh37 with backbone resolution ~50 Kb and targeted gene average resolution ~10Kb. CN was determined by the signal intensity of all 2.67 million markers and genotyping was assessed by signal intensities for the A and B alleles, yielding allelic values.

*Copy Number Abnormalities (CNA) detection.* CNA detection was performed using an in-house developed algorithm (*n* = 72) as previously described [2,9].

*Copy neutral loss of heterozygosity (cnLOH) detection.* Where applicable, patient samples with no copy number abnormalities were checked for evidence of cnLOH by informative SNPs in genes either side of the *TP53* gene from available NGS data.

**Next-generation sequencing panels.** DNA was extracted from bone marrow aspirates and sequencing was performed using a targeted next-generation sequencing (NGS) panel at each institution. Frequently mutated genes in myeloid malignancies were sequenced and analyzed: *ASXL1, BCOR, CBL, CEBPA, DDX41, DNMT3A, EZH2, FLT3, GATA2, IDH1, IDH2, JAK2, KIT, KRAS, MPL, NPM1, NRAS, PTPN11, RUNX1, SETBP1, SF3B1, SRP72, SRSF2, STAG2, TERT, TET2, TP53, U2AF1, WT1* and *ZRSR2.*

*Mayo Clinic* *NGS panel*. The library preparation, sequencing and data analysis were performed as described [10]. Briefly, libraries were prepared using the Agilent SureSelect‐XT Target Enrichment Kit (SureSelectXT, Agilent, Santa Clara, CA) and sequencing was performed on MiSeq or HiSeq platforms (Illumina, San Diego, CA) at the Mayo Clinic Clinical Genome Sequencing Laboratory.

*South Australian NGS panel.* The library preparation, sequencing and data analysis were performed as described [11]. Briefly, libraries were prepared using NimbleGen Capture Platform (Roche NimbleGen Inc., Madison, WI, USA) and sequenced on an Illumina HiSeq2500 sequencing system (Illumina). Entire coding regions were sequences for each gene. Only variants with a total read depth >50, supported by more than five alternate variant reads and a variant allele frequency (VAF) ≥2%, were retained for further analysis. Pathogenic and likely pathogenic variants calling was performed as described [11].

**Variant annotation for oncogenicity.** Somatic variants were classed as oncogenic as previously described [2,12].

**Annotation of *TP53* mutations.** *TP53* mutations (*TP53*^mut^) were classified as missense, splice-site and truncated mutations which included frameshift indels or nonsense mutations. Missense mutations at amino acid positions 175, 220, 237, 248, 273 and 282 were annotated as hotspots [13,14].

*TP53*^mut^ were classed as single-hit by the presence of a single gene mutation of VAF 10% - 50% without loss of *TP53* locus, or loss of *TP53* locus without *TP53*^mut^. Multi-hit *TP53*^mut^ were determined by the presence of two or more distinct *TP53*^mut^ (VAF ≥10%), or a single *TP53*^mut^ associated with either: i) A cytogenetic deletion involving the *TP53* locus at 17p13.1; ii) A VAF of >50%; or iii) Copy-neutral loss of heterozygosity (cnLOH) at the 17p *TP53* locus [15]. Loss of 17p was defined by allelic imbalances leading to loss of *TP53* (del 17p, monosomy 17, isochromosome 17q and 17p translocation/dicentric/derivative events identified as having a chromosomal 17 break at segment p11.2).

**Statistical analysis.** Fisher’s exact test was used to compare categorical variables. Wilcoxon rank-sum test or Student’s t test were used to compare continuous variables. All statistical tests were two-sided. Multiple testing correction was applied when appropriate using the Benjamini–Hochberg method. Overall survival (OS) was calculated from date of t-MN diagnosis to time of death (from any cause). OS for transplant was assessed from day of transplantation. Patients alive at the last follow-up date were censored. Survival probabilities over time were estimated using Kaplan–Meier methodology, and comparisons of survival across subgroups were conducted using the two-sided log-rank test. Kaplan–Meier estimates were computed using the R package survival. Optimal cut-point for *TP53* VAF was determined using the maximally selected rank statistics from the “maxstat” R package [16]. Multivariable models of overall survival were performed with Cox proportional hazards regression, using the R package coxph. Hazard ratios and 95% confidence interval (CI) were reported for covariates, along with *P* values from the Wald test. Covariates included in the multivariable model of overall survival are derived from statistically significance from univariate and based on backward-selection approach. Allogeneic SCT was entered as time-dependent variable in the multivariate analysis. *P* values <0.05 were considered statistically significant. Survival contour plot was generated using *contsurvplot* R package to visualize the causal effect of *TP53* VAF on a time-to-event OS outcome. All statistical analyses were conducted using the R statistical platform (https://www.r-project.org/) v.4.1.1.

**Supplementary Tables**

**Table S1: Clinical features of t-MN**

| Variables | Whole cohort  (*n* = 488) |
| --- | --- |
| Clinical features at t-MN diagnosis | |
| Age at t-MN diagnosis, median (IQR) | 68.0 (60.4, 74.6) |
| Female/male, n | 203/285 |
| Hb g/dL, median (IQR) | 9.4 (8.1, 11.1) |
| WBC x 10^9^/L, median (IQR) | 3.40 (2.10, 5.90) |
| ANC x 10^9^/L, median (IQR) | 1.12 (0.49, 2.38) |
| Platelets x 10^9^/L, median (IQR) | 67.0 (37.0, 115.0) |
| BM blasts, n (%) <5% 5-9% 10-19% ≥20% | 190 (39.1) 68 (14.0) 63 (13.0)  165 (34.0) |
| Cytogenetic changes | |
| Number of cases with cytogenetic aberrancy, n (%) | 365 (75.7) |
| Complex karyotype, n (%) | 190 (39.3) |
| Monosomal karyotype, n (%) | 183 (37.9) |
| Marker chromosome, n (%) | 113 (23.5) |
| Ring chromosome, n (%) | 48 (10.0) |
| Abnormal Chrom. 7, n (%) | 148 (30.6) |
| Abnormal Chrom. 5, n (%) | 108 (22.4) |
| Abnormal Chrom. 11, n (%) | 82 (17.0) |
| Abnormal Chrom. 12, n (%) | 67 (13.9) |
| Abnormal Chrom. 3, n (%) | 71 (14.7) |
| Abnormal Chrom. 21, n (%) | 77 (15.9) |
| Abnormal Chrom. 13, n (%) | 66 (13.7) |
| Abnormal Chrom. 9, n (%) | 60 (12.4) |
| Abnormal Chrom. 18, n (%) | 62 (12.8) |
| Trisomy 8, n (%) | 65 (13.5) |
| Abnormal Chrom. 20, n (%) | 67 (13.9) |
| Abnormal Chrom. 19, n (%) | 55 (11.4) |
| Abnormal Chrom. 16, n (%) | 56 (11.6) |
| Abnormal Chrom. 17, n (%) | 58 (12.0) |
| Number of co-mutations  ≥ 2 mutations, n (%) 1 mutation, n (%)  No mutation, n (%) | 313 (64.1) 121 (24.8) 54 (11.1) |
| *TP53* mutation ± 17p13 loss VAF, median (IQR)  VAF <10%  VAF ≥10%  Single-hit  Multi-hit | **182 (37.3%)**  36.9 (20.6, 47.2)  20 (11.2)  158 (88.7)  34 (19.9)  137 (80.1) |
| *ASXL1* mutation, n (%) | 83 (17.0) |
| *TET2* mutations, n (%) | 82 (16.8) |
| *DNMT3A* mutations, n (%) | 79 (16.2) |
| *SRSF2* mutations, n (%) | 59 (12.1) |
| *RAS* mutations, n (%) | 49 (10.1) |
| *RUNX1* mutations, n (%) | 55 (11.3) |
| *BCOR* mutations, n (%) | 28 (5.8) |
| *IDH2* mutations, n (%) | 27 (5.5) |
| *WT1* mutations, n (%) | 19 (4.1) |
| *SF3B1* mutations, n (%) | 26 (5.3) |
| *U2AF1* mutations, n (%) | 26 (5.4) |
| *EZH2* mutations, n (%) | 18 (3.7) |
| *NPM1* mutations, n (%) | 20 (4.1) |
| *STAG2* mutations, n (%) | 20 (4.1) |
| *IDH1* mutations, n (%) | 18 (3.7) |
| *FLT3-ITD* mutations, n (%) | 18 (4.6) |
| *PTPN11* mutations, n (%) | 18 (3.7) |
| *SETBP1* mutations, n (%) | 15 (3.1) |
| *GATA2* mutations, n (%) | 12 (2.5) |
| *FLT3*-*TKD* mutations, n (%) | 10 (2.6) |
| *JAK2* mutations, n (%) | 13 (2.7) |
| *TERT* mutations, n (%) | 10 (2.3) |
| *CBL* mutations, n (%) | 10 (2.1) |
| *KIT* mutations, n (%) | 9 (1.8) |
| *ZRSR2* mutations, n (%) | 8 (1.7) |
| *DDX41* mutations, n (%) | 5 (1.0) |
| *MPL* mutations, n (%) | 5 (1.0) |
| Disease modifying therapy for t-MN*  No DMT, n (%) Intensive chemotherapy, n (%) HMA based chemotherapy, n (%) Venetoclax based therapy, n (%) | 138 (28.3) 100 (20.5) 160 (32.8)  70 (14.3) |
| Allogeneic SCT, n (%) | 91 (18.6) |
| Interval between primary to t-MN (months), median (IQR) | 81.10 (40.7, 149.4) |
| Clinical features at primary disease |  |
| Age at primary disease (years), median (IQR) | 59 (50, 66) |
| Hematology malignancy, n (%) Solid cancer, n (%)  AID, n (%)  Hem and solid cancer, n (%)  Two independent solid cancers, n (%)  Solid cancer and AID, n (%) Hem malignancy and AID, n (%)  Two independent hem malignancies, n (%)  Other, n (%) | 225 (46.1) 179 (36.7)  33 (6.8) 22 (4.5)  17 (3.5)  6 (1.2)  3 (0.6)  1 (0.2)  2 (0.4) |
| Chemotherapy for primary cancer/disease | 230 (47.1) |
| Chemotherapy plus radiotherapy for primary cancer/disease | 160 (32.8) |
| Radiation alone for primary cancer, n (%) | 79 (16.2) |
| Auto SCT for primary cancer, n (%) | 95 (19.5) |
| Immunosuppression, n (%) | 53 (10.9) |

Cytogenetics data was unavailable in 5 patients, BM blast % was unknown in 2 patients.

Abbreviations: t-MN – therapy related myeloid neoplasm; Hb – Hemoglobin; WBC – white blood cell; ANC – absolute neutrophil count; BM – Bone marrow; Chrom. – Chromosome; VAF – Variant Allele Frequency; DMT – disease-modifying therapy; HMA – hypomethylating agents; AID – Autoimmune disease; SCT – Stem Cell Transplant.

*First line of therapy only

**Table S2:** **Comparison of clinical features of *TP53*^wt^ versus *TP53*^mut^ ≥ 10% OR loss of *TP53* locus without *TP53*^mut^ or VAF <10%**

| Variables | *TP53*^mut^ ≥ 10% OR loss of *TP53* locus without *TP53*^mut^ or VAF <10%  (*n* = 171) | *TP53*^wt^  (*n* = 298) | *P*-value |
| --- | --- | --- | --- |
| Clinical features at t-MN diagnosis | | | |
| Age at t-MN diagnosis, median (IQR) | 68.3 (61.9, 74.4) | 67.9 (59.0,74.9) | 0.509 |
| Female/male | 66/105 | 129/169 | 0.332 |
| Hb g/dL, median (IQR) | 9.0 (8.0, 10.3) | 9.7 (8.3, 11.3) | **<0.001** |
| WBC x 10^9^/L, median (IQR) | 2.80 (1.80, 4.20) | 3.65 (2.21, 8.92) | **<0.001** |
| ANC x 10^9^/L, median (IQR) | 0.87 (0.49, 1.88) | 1.23 (0.49, 3.07) | **0.01** |
| Platelets x 10^9^/L, median (IQR) | 55.0 (29.0, 97.0) | 71.0 (41.0, 134.0) | **<0.001** |
| BM blasts, n (%)  <5%  5-9%  10-19%  ≥20% | 71 (41.8)  26 (15.3)  24 (14.1)  49 (28.8) | 110 (37.0)  36 (12.1)  38 (12.8)  113 (38.0) | 0.229 |
| Disease phenotype  t-MDS  t-AML | 122 (71.3)  49 (28.7) | 180 (60.4)  118 (39.6) | 0.021 |
| Cytogenetic changes | | | |
| Number of cases with cytogenetic aberrancies, n (%) | 166 (97.1) | 189 (64.1) | **<0.001** |
| Complex karyotype, n (%) | 146 (85.4) | 36 (12.2) | **<0.001** |
| Monosomal karyotype, n (%) | 137 (80.1) | 39 (13.2) | **<0.001** |
| Marker chromosome, n (%) | 137 (80.1) | 39 (13.2) | **<0.001** |
| Ring chromosome, n (%) | 40 (23.5) | 5 (1.7) | **<0.001** |
| Abnormal Chrom. 17, n (%) | 58 (33.9) | 0 (0.0) | **<0.001** |
| Del 5q/ Monosomy 5, n (%) | 93 (54.4) | 12 (4.1) | **<0.001** |
| Del 7q/ Monosomy 7, n (%) | 83 (48.5) | 59 (20.0) | **<0.001** |
| Abnormal Chrom. 3, n (%) | 37 (21.6) | 26 (8.8) | **<0.001** |
| Trisomy 8, n (%) | 35 (20.5) | 22 (7.5) | **<0.001** |
| Abnormal Chrom. 9, n (%) | 35 (20.5) | 22 (7.5) | **<0.001** |
| Abnormal Chrom. 11, n (%) | 45 (26.3) | 34 (11.5) | **<0.001** |
| Abnormal Chrom. 12, n (%) | 54 (31.6) | 10 (3.4) | **<0.001** |
| Abnormal Chrom. 13, n (%) | 54 (31.6) | 10 (3.4) | **<0.001** |
| Abnormal Chrom. 16, n (%) | 41 (24.0) | 14 (4.7) | **<0.001** |
| Abnormal Chrom. 18, n (%) | 55 (32.2) | 4 (1.4) | **<0.001** |
| Abnormal Chrom. 19, n (%) | 40 (23.4) | 14 (4.7) | **<0.001** |
| Abnormal Chrom. 20, n (%) | 43 (25.1) | 23 (7.8) | **<0.001** |
| Abnormal Chrom. 21, n (%) | 57 (33.3) | 20 (6.8) | **<0.001** |
| Somatic mutations on NGS |  |  |  |
| *TP53* mutation *±* 17p13 loss Single-hit, n (%) Multi-hit, n (%) VAF, median (IQR) | 34 (19.9) 137 (80.1)  37.3 (25.0-49.2) | NA | **NA** |
| Co-mutations excluding *TP53*  ≥ 2 mutations, n (%)  1 mutation, n (%)  No mutation, n (%) | 25 (14.6)  42 (24.6)  104 (60.8) | 193 (64.8)  52 (17.4)  53(17.8) | **<0.001** |
| *DNMT3A* mutations, n (%) | 20 (11.7) | 57 (19.1) | **0.039** |
| *RAS* mutations, n (%) | 4 (2.3) | 43 (14.4) | **<0.001** |
| *TET2* mutations, n (%) | 12 (7.0) | 65 (21.8) | **<0.001** |
| *ASXL1* mutations*,* n (%) | 11 (6.4) | 70 (23.5) | **<0.001** |
| *RUNX1* mutations, n (%) | 8 (4.7) | 45 (15.1) | **<0.001** |
| *SF3B1* mutations, n (%) | 5 (2.9) | 20 (6.7) | 0.09 |
| *SRSF2* mutations, n (%) | 7 (4.1) | 51 (17.1) | **<0.001** |
| *IDH2* mutations, n (%) | 1 (0.6) | 26 (8.7) | **<0.001** |
| *FLT3* mutations, n (%) | 2 (1.2) | 27 (9.1) | **<0.001** |
| Disease modifying therapy for t-MN* |  |  |  |
| No DMT Intensive chemotherapy HMA based chemotherapy Venetoclax based therapy | 32 (18.7) 20 (11.7) 74 (43.3) 39 (22.8) | 100 (33.6) 76 (25.5) 78 (26.2) 30 (10.1) | **<0.0001** |
| Allogeneic SCT, n (%) | 24 (14.0) | 63 (21.1) | 0.064 |
| Interval between primary to t-MN (months), median (IQR) | 89.3 (48.1, 154.1) | 73.8 (37.1, 141.9) | **0.015** |
| Clinical features at primary disease |  |  |  |
| Age at primary disease, median (IQR) | 58 (50.0, 66.0) | 59.0 (49.2, 66.0) | 0.904 |
| Hematology malignancy, n (%)  Solid cancer, n (%)  AID, n (%)  Hem and solid cancer, n (%)  Two independent solid cancers, n (%)  Solid cancer and AID, n (%)  Hem malignancy and AID, n (%)  Two independent hem malignancy n (%)  Other, n (%) | 93 (54.4) 48 (28.1) 11 (6.4)  8 (4.7)  8 (4.7)  2 (1.2)  0 (0.0)  1 (0.6)  1 (0.6) | 121 (40.6) 125 (41.9) 21 (7.0)  14 (4.7)  9 (3.0)  4 (1.3)  3 (1.0)  0 (0.0)  0 (0.0) | **0.039** |
| Chemotherapy for primary cancer/disease, n (%) | 83 (48.5) | 136 (45.6) | 0.565 |
| Radiation for primary cancer, n (%) | 16 (9.4) | 61 (20.5) | **0.002** |
| Auto SCT for primary cancer, n (%) | 41 (24.0) | 48 (16.1) | **0.038** |
| Immunosuppression, n (%) | 17 (9.9) | 34 (11.4) | 0.758 |

*First line of therapy only

**Table S3: Cox regression univariate analysis of variables predicting OS in the whole t-MN cohort**

| Variables | HR | *P*-value |
| --- | --- | --- |
| Clinical features at t-MN diagnosis |  |  |
| Age at t-MN diagnosis | 1.02 (1.01-1.03) | **0.00013** |
| Gender (Reference - Female) | 1.05 (0.83-1.31) | 0.688 |
| Hb g/dL at t-MN diagnosis | 0.85 (0.81-0.90) | **<0.0001** |
| WBC x10^9^/L at t-MN diagnosis | 1 (0.99 to 1.01) | 0.31 |
| ANC x10^9^/L at t-MN diagnosis | 1.01 (0.99-1.02) | 0.117 |
| Platelets x10^9^/L at t-MN diagnosis | 0.998 (0.997-0.999) | **0.0004** |
| BM blast (Reference - <5%) |  |  |
| BM blast 5-9% | 1.49 (1.08-2.08) | **0.016** |
| BM blast 10-19% | 1.67 (1.19-2.35) | **0.003** |
| BM blast ≥20% | 1.71 (1.31-2.22) | **<0.0001** |
| *TP53*^mut^ | 2.64 (2.1-3.33) | **<0.0001** |
| Cytogenetic changes |  |  |
| Complex karyotype | 1.96 (1.57-2.45) | **<0.0001** |
| Del 5q/ Monosomy 5 | 2.02 (1.57-2.59) | **<0.0001** |
| Complex and monosomal karyotype | 2.13 (1.69-2.68) | **<0.0001** |
| Monosomal karyotype | 2.26 (1.81-2.83) | **<0.0001** |
| Abnormal Chrom. 17 | 2.47 (1.9-3.2) | **<0.0001** |
| Abnormal Chrom. 18 | 2.66 (1.98-3.56) | **<0.0001** |
| Abnormal Chrom. 13 | 2.09 (1.56-2.79) | **<0.0001** |
| Marker chromosome | 2.0 (1.57-2.55) | **<0.0001** |
| Abnormal Chrom. 19 | 2.09 (1.51-2.89) | **<0.0001** |
| Abnormal Chrom. 21 | 1.97 (1.5-2.6) | **<0.0001** |
| Abnormal Chrom. 3 | 1.85 (1.39-2.45) | **0.002** |
| Abnormal Chrom. 12 | 1.64 (1.23-2.2) | **0.0008** |
| Del 7q/ Monosomy 7 | 1.55 (1.25-1.94) | **<0.0001** |
| Abnormal Chrom. 11 | 1.62 (1.23-2.15) | **0.0006** |
| Abnormal Chrom. 16 | 1.71 (1.23-2.39) | **0.001** |
| Trisomy Chrom. 8 | 1.50 (1.11-2.04) | **0.008** |
| Abnormal Chrom. 20 | 1.56 (1.16-2.1) | **0.003** |
| Ring chromosome | 1.93 (1.38-2.69) | **0.0001** |
| Deletion Chrom. Y | 1.54 (0.95-2.48) | 0.073 |
| Abnormal Chrom. 9 | 1.28 (0.93-1.77) | 0.128 |
| Number of mutations (Ref - No mutation) | 1.66 (1.09-2.54) | 0.019 |
| *IDH2* mutations | 0.51 (0.28-0.94) | 0.031 |
| *TERT* mutations | 0.39 (0.16-0.96) | 0.041 |
| *SF3B1* mutations | 0.57 (0.33-0.98) | 0.044 |
| Treatment of t-MN (Reference - Supportive care) |  |  |
| Intensive chemotherapy | 0.79 (0.57-1.09) | 0.155 |
| HMA based therapy | 1.15 (0.88-1.50) | 0.307 |
| Venetoclax based therapies | 1.30 (0.89-1.88) | 0.167 |
| Allogeneic SCT (Reference - No) | 0.329 (0.23-0.46) | **<0.0001** |
| Clinical features at primary disease |  |  |
| Age at primary disease, median (IQR) | 1.02 (1.01-1.03) | **<0.0001** |

**Table S4: Comparison of clinical features in *TP53*^mut^ variant allele frequency (VAF) <10% with *TP53*^wt^**

| Variables | *TP53*^mut^ VAF <10%  (*n =* 15) | *TP53*^wt^  (*n =* 298) | *P*-value |
| --- | --- | --- | --- |
| Clinical features at t-MN diagnosis | | | |
| Age at t-MN diagnosis, median (IQR) | 67.0 (56.5-75.0) | 67.9 (59.0, 74.9) | 0.828 |
| Female/male | 7/8 | 129/169 | 0.796 |
| Hb g/dL, median (IQR) | 9.70 (8.25,11.7) | 9.70 (8.30, 11.3) | 0.763 |
| WBC x 10^9^/L, median (IQR) | 3.81 (2.67,5.05) | 3.65 (2.21, 8.92) | 0.794 |
| ANC x 10^9^/L, median (IQR) | 1.69 (0.40,2.74) | 1.23 (0.49, 3.07) | 0.889 |
| Platelets x 10^9^/L, median (IQR) | 82.0 (50.5, 110.5) | 71.0 (41.0, 134.0) | 0.747 |
| BM blasts %, n (%)  <5%  5-9%  10-19%  ≥20% | 8 (53.3)  5 (33.3)  0 (0.0)  2 (13.3) | 110 (37.0)  36 (12.1)  38 (12.8)  113 (38.0) | **0.0017** |
| Cytogenetic changes | | | |
| Number of cases with cytogenetic aberrancy, n (%) | 7 (53.8) | 189 (64.1) | 0.558 |
| Complex karyotype, n (%) | 5 (35.7) | 36 (12.2) | **0.026** |
| Monosomal karyotype, n (%) | 4 (28.6) | 39 (13.2) | 0.114 |
| Del 5q/ Monosomy 5, n (%) | 3 (21.4) | 12 (4.1) | **0.024** |
| Marker chromosome, n (%) | 3 (23.1) | 18 (6.1) | **0.05** |
| Del 7q/ Monosomy 7, n (%) | 3 (21.4) | 59 (20.0) | 1 |
| Abnormal Chrom. 21, n (%) | 0 (0.0) | 20 (6.8) | 0.611 |
| Ring chromosome, n (%) | 3 (23.1) | 5 (1.7) | **0.003** |
| Somatic mutations on NGS | | | |
| Co-mutations excluding *TP53*  ≥ 2 mutations, n (%)  1 mutation, n (%)  No mutation, n (%) | 6 (40.0)  2 (13.3)  7 (46.7) | 193 (64.8)  52 (17.4)  53 (17.8) | 0.028 |
| *DNMT3A* mutations, n (%) | 1 (6.7) | 57 (19.1) | 0.32 |
| *RAS* mutations, n (%) | 2 (13.3) | 43 (14.4) | 1 |
| *TET2* mutations, n (%) | 5 (33.3) | 65 (21.8) | 0.339 |
| Clinical features at primary disease | | | |
| Age at primary disease, median (IQR) | 57.0 (48.5,68.7) | 59.0 (49.2, 66.0) | 0.928 |
| Hematology malignancy, n (%)  Solid cancer, n (%)  AID, n (%)  Hem and solid cancer, n (%)  Two independent solid cancers, n (%)  Solid cancer and AID, n (%)  Hem malignancy and AID, n (%)  Two independent hem malignancy n (%) | 8 (53.3)  6 (40.0)  1 (6.7)  0 (0.0)  0 (0.0)  0 (0.0)  0 (0.0)  0 (0.0) | 121 (40.6)  125 (41.9)  21 (7.0)  14 (4.7)  9 (3.0)  4 (1.3)  3 (1.0)  1 (0.3) | 0.969 |
| Chemotherapy for primary cancer/disease, n (%) | 8 (53.3) | 136 (45.6) | 0.604 |
| Radiation for primary cancer, n (%) | 2 (13.3) | 61 (20.5) | 0.744 |
| Auto SCT for primary cancer, n (%) | 4 (26.7) | 48 (16.1) | 0.287 |
| Immunosuppression, n (%) | 1 (6.7) | 34 (11.4) | 1 |

**Table S5: Comparison of clinical features in *TP53*^mut^ variant allele frequency (VAF) <10% with *TP53*^mut^ VAF ≥10% with or without loss of *TP53* locus**

| Variables | VAF <10%  (*n =* 15) | VAF $\boldsymbol{\geq}$10% ± *TP53* loss  (*n =* 171) | *P*-value |
| --- | --- | --- | --- |
| Clinical features at t-MN diagnosis |  |  |  |
| Age at t-MN diagnosis, median (IQR) | 67.0 (56.5, 75.0) | 68.3 (61.9, 74.4) | 0.647 |
| Female/male | 7/8 | 66/105 | 0.588 |
| Hb g/dL, median (IQR) | 9.70 (8.25,11.7) | 9.0 (8.0, 10.3) | 0.131 |
| WBC x 10^9^/L, median (IQR) | 3.81 (2.67, 5.05) | 2.80 (1.80, 4.20) | 0.105 |
| ANC x 10^9^/L, median (IQR) | 1.69 (0.40, 2.74) | 0.87 (0.49, 1.88) | 0.243 |
| Platelets x 10^9^/L, median (IQR) | 82.0 (50.5,110.5) | 55.0 (29.0, 97.0) | 0.07 |
| BM blasts %, n (%)  <5%  5-9%  10-19%  ≥20% | 8 (53.3)  5 (33.3)  0 (0.0)  2 (13.3) | 71 (41.8)  26 (15.3)  24 (14.1)  49 (28.8) | 0.089 |
| Cytogenetics | | | |
| Monosomal karyotype, n (%) | 4 (28.6) | 137 (80.1) | **<0.001** |
| Complex karyotype, n (%) | 5 (35.7) | 146 (85.4) | **<0.001** |
| Marker chromosome, n (%) | 3 (23.1) | 90 (52.9) | **0.046** |
| Del 5q/ Monosomy 5 | 3 (21.4) | 93 (54.4) | **0.024** |
| Abnormal Chrom. 17, n (%) | 0 (0.0) | 58 (33.9) | **0.006** |
| Del 7q/ Monosomy 7 | 3 (21.4) | 83 (48.5) | 0.056 |
| Ring chromosome, n (%) | 3 (23.1) | 40 (23.5) | 1 |
| *TP53* mutation ± 17p13 loss VAF median (IQR) | 5.3 (2.4, 7.0) | 37.3 (25.0, 49.2) | **<0.0001** |
| Somatic mutations on NGS |  |  |  |
| Co-mutations excluding *TP53*  ≥ 2 mutations, n (%)  1 mutation, n (%)  No mutation, n (%) | 6 (40.0)  2 (13.3)  7 (46.7) | 25 (14.6)  42 (24.6)  104 (60.8) | 0.063 |
| *TET2* mutations, n (%) | 5 (33.3) | 12 (7.0) | **0.006** |
| *DNMT3A* mutations, n (%) | 1 (6.7) | 20 (11.7) | 1 |
| *RAS* mutations, n (%) | 2 (13.3) | 4 (2.3) | 0.076 |
| *ASXL1* mutations, n (%) | 2 (13.3) | 11 (6.4) | 0.282 |
| *RUNX1* mutations*,* n (%) | 2 (13.3) | 8 (4.7) | 0.187 |
| *SF3B1* mutations, n (%) | 1 (6.7) | 5 (2.9) | 0.401 |
| *SRSF2* mutations, n (%) | 1 (6.7) | 7 (4.1) | 0.497 |
| Disease modifying therapy for t-MN* | | | |
| No DMT Intensive chemotherapy HMA based chemotherapy Venetoclax based therapy | 6 (40.0) 3 (20.0) 5 (33.3) 1 (6.7) | 32 (18.2) 20 (11.7) 74 (43.3) 39 (22.8) | 0.314 |
| Allogeneic SCT, n (%) | 4 (26.7) | 24 (14.0) | 0.249 |
| Interval between primary to t-MN (months), median (IQR) | 68.8 (32.3, 141.8) | 89.3 (48.1, 154.1) | 0.276 |
| Clinical features at primary disease |  |  |  |
| Age at primary disease, median (IQR) | 57.0 (48.5, 68.7) | 58.0 (50.0, 66.0) | 0.959 |
| Hematology malignancy, n (%)  Solid cancer, n (%)  AID, n (%)  Hem and solid cancer, n (%)  Two independent solid cancers, n (%)  Solid cancer and AID, n (%)  Other, n (%) | 8 (53.3)  6 (40.0)  1 (6.7)  0 (0.0)  0 (0.0)  0 (0.0) 0 (0.0) | 93 (54.4)  48 (28.1)  11 (6.4)  8 (4.7)  8 (4.7)  2 (1.2) 1 (0.6) | 0.941 |
| Chemotherapy for primary cancer/disease | 08 (53.3) | 83 (48.5) | 0.792 |
| Radiation for primary cancer, n (%) | 2 (13.3) | 16 (9.4) | 0.643 |
| Auto SCT for primary cancer, n (%) | 4 (26.7) | 41 (24.0) | 0.761 |
| Immunosuppression, n (%) | 1 (6.7) | 17 (9.9) | 1 |

*First line of therapy only

**Table S6: Comparison of genomic instability and other clinical features in single-hit and multi-hit *TP53* mutated t-MN**

| Variables | Multi-hit  (*n =* 137) | Single-hit  (*n =* 34) | *P*-value |
| --- | --- | --- | --- |
| Clinical features at t-MN diagnosis | | | |
| Age at t-MN diagnosis, median (IQR) | 68 (62, 74) | 69 (62, 75) | 0.64 |
| Female/male | 56/81 | 10/24 | 0.244 |
| Hb g/dL, median (IQR) | 9.0 (7.80, 10.50) | 8.60 (8.00, 10.20) | 0.455 |
| WBC x 10^9^/L, median (IQR) | 2.80 (1.69, 4.17) | 2.86 (2.16, 5.22) | 0.321 |
| ANC x 10^9^/L, median (IQR) | 0.79 (0.50, 1.82) | 1.15 (0.47, 2.18) | 0.559 |
| Platelets x 10^9^/L, median (IQR) | 61.0 (30.0, 100.2) | 38.0 (28.0, 78.0) | 0.116 |
| BM blasts %, n (%)  <5%  5-9%  10-19%  ≥20% | 54 (39.4)  23 (16.8)  21 (15.3)  39 (28.5) | 17 (51.5)  3 (9.1)  3 (9.1)  10 (30.3) | 0.508 |
| t-MN phenotype  t-MDS  t-AML | 98 (71.5)  39 (28.5) | 24 (70.6)  10 (29.4) | 1 |
| Cytogenetic changes | | | |
| Any cytogenetic aberrancies | 135 (98.5) | 31 (91.2) | 0.054 |
| Complex karyotype | 121 (88.3) | 25 (73.5) | 0.053 |
| Monosomal karyotype, n (%) | 113 (82.5) | 24 (70.6) | 0.149 |
| Marker chromosome, n (%) | 78 (57.4) | 12 (35.3) | **0.034** |
| Ring chromosome, n (%) | 35 (25.7) | 5 (14.7) | 0.258 |
| Abnormal Chrom. 17, n (%) | 45 (32.8) | 13 (38.2) | 0.551 |
| Del 5q/ Monosomy 5, n (%) | 79 (57.7) | 14 (41.2) | 0.123 |
| Del 7q/ Monosomy 7, n (%) | 66 (48.2) | 17 (50.0) | 0.851 |
| Abnormal Chrom. 3, n (%) | 41 (29.9) | 7 (20.6) | 0.394 |
| Trisomy 8, n (%) | 31 (22.6) | 6 (17.6) | 0.645 |
| Abnormal Chrom. 9, n (%) | 29 (21.2) | 6 (17.6) | 0.813 |
| Abnormal Chrom. 11, n (%) | 38 (27.7) | 7 (20.6) | 0.515 |
| Abnormal Chrom. 12, n (%) | 46 (33.6) | 8 (23.5) | 0.307 |
| Abnormal Chrom. 13, n (%) | 46 (33.6) | 8 (23.5) | 0.307 |
| Abnormal Chrom. 16, n (%) | 35 (25.5) | 6 (17.6) | 0.379 |
| Abnormal Chrom. 18, n (%) | 42 (30.7) | 13 (38.2) | 0.417 |
| Abnormal Chrom. 19, n (%) | 33 (24.1) | 7 (20.6) | 0.822 |
| Abnormal Chrom. 20, n (%) | 37 (27.0) | 6 (17.6) | 0.377 |
| Abnormal Chrom. 21, n (%) | 48 (35.0) | 9 (26.5) | 0.419 |
| Somatic mutations on NGS | | | |
| *TP53* mutation ± 17p13 loss VAF, median (IQR) | 38.2 (27.0, 54.4) | 34.0 (9.5, 42.0) | **0.006** |
| Co-mutations excluding *TP53*  ≥2 mutations (n, %)  1 mutation (n, %)  No mutation (n, %) | 17 (12.4)  32 (23.4)  88 (64.2) | 8(23.5)  10 (29.4)  16 (47.1) | 0.123 |
| *DNMT3A* mutations, n (%) | 13 (9.5) | 7 (20.6) | 0.08 |
| *RAS* mutations, n (%) | 2 (1.5) | 2 (5.9) | 0.177 |
| *TET2* mutations, n (%) | 9 (6.6) | 3 (8.8) | 0.707 |
| *ASXL1* mutations*,* n (%) | 8 (5.8) | 3 (8.8) | 0.459 |
| *RUNX1* mutations, n (%) | 6 (4.4) | 2 (5.9) | 0.659 |
| *SF3B1* mutations*,* n (%) | 3 (2.2) | 2 (5.9) | 0.259 |
| *SRSF2* mutations*,* n (%) | 5 (3.6) | 2 (5.9) | 0.627 |
| Disease modifying therapy for t-MN* | | | |
| No DMT Intensive chemotherapy HMA based chemotherapy Venetoclax based therapy | 24 (17.5)  16 (11.7)  58 (42.3)  36 (26.3) | 8 (23.5)  4 (11.8)  16 (47.1)  3 (8.8) | 0.082 |
| Allogeneic SCT, n (%) | 21 (15.3) | 3 (8.8) | 0.418 |
| Months between primary to t-MN, median (IQR) | 90.2 (47.9, 154.5) | 75.6 (49.6, 149.2) | 0.813 |
| Clinical features at primary disease | | | |
| Age at primary disease, median (IQR) | 58.0 (50.0, 66.0) | 58.5 (51.0, 65.0) | 0.965 |
| Hematology malignancy, n (%)  Solid cancer, n (%)  AID, n (%)  Hem and solid cancer, n (%)  Two independent solid cancers, n (%)  Solid cancer and AID, n (%)  Other, n (%) | 76 (55.5)  36 (26.3)  9 (6.6)  5 (3.6)  8 (5.8)  2 (1.5)  1 (0.7) | 17 (50.0)  12 (35.3)  2 (5.9)  3 (8.8)  0 (0.0)  0 (0.0)  0 (0.0) | 0.5125 |
| Chemotherapy for primary cancer/disease | 69 (50.4) | 14 (41.2) | 0.444 |
| Radiation for primary cancer, n (%) | 13 (9.5) | 3 (8.8) | 1 |
| Auto SCT for primary cancer, n (%) | 31 (22.6) | 10 (29.4) | 0.501 |
| Immunosuppression, n (%) | 15 (10.9) | 2 (5.9) | 0.53 |

*First line of therapy only

**Table S7: Comparison of clinical features and genomic instability in complex karyotype t-MN with and without *TP53*^mut^**

| Variables | *TP53*^wt^-CK  (*n =* 36) | *TP53*^mut^-CK  (*n* = 146) | *P*-value |
| --- | --- | --- | --- |
| Clinical features at t-MN diagnosis |  |  |  |
| Age at t-MN diagnosis, median (IQR) | 66.3 (47.6, 73.1) | 68.2 (62.1, 74.6) | 0.064 |
| Female/male | 17/19 | 55/91 | 0.343 |
| Hb g/dL, median (IQR) | 9.95 (8.10,12.07) | 9.00(8.00, 10.35) | **0.035** |
| WBC x 10^9^/L, median (IQR) | 3.60 (2.30, 6.60) | 2.84 (1.84, 4.20) | **0.033** |
| ANC x 10^9^/L, median (IQR) | 1.40 (0.40, 2.11) | 0.87 (0.50, 1.88) | 0.375 |
| Platelets x 10^9^/L, median (IQR) | 64.0 (41.0, 89.0) | 57.0 (30.0, 94.7) | 0.694 |
| BM blasts %, n (%)  <5%  5-9%  10-19%  ≥20% | 16 (44.4)  4 (11.1)  5 (13.9)  11 (30.6) | 61 (41.8)  23 (15.8)  24 (16.4)  38 (26.0) | 0.881 |
| Cytogenetics | | | |
| Monosomal karyotype, n (%) | 18 (50.0) | 124 (84.9) | **<0.001** |
| Marker chromosome, n (%) | 11 (30.6) | 85 (58.6) | **0.003** |
| Abnormal Chrom. 17, n (%) | 0 (0.0) | 51 (34.9) | **<0.001** |
| Del 5q/ Monosomy 5, n (%) | 6 (16.7) | 83 (56.8) | **<0.001** |
| Abnormal Chrom. 12, n (%) | 5 (13.9) | 52 (35.6) | **0.015** |
| Abnormal Chrom. 18, n (%) | 3 (8.3) | 51 (34.9) | **0.001** |
| Del 7q/ Monosomy 7, n (%) | 13 (36.1) | 73 (50.0) | 0.142 |
| Ring chromosome, n (%) | 1 (2.8) | 28 (26.2) | **0.001** |
| Co-mutations excluding *TP53*  ≥ 2 mutations, n (%)  1 mutation, n (%)  No mutation, n (%) | 15 (41.7) 7 (19.4)  14 (38.9) | 16 (11.0)  40 (27.4)  90 (61.6) | **<0.001** |
| *DNMT3A* mutations, n (%) | 5 (13.9) | 15 (10.3) | 0.554 |
| *RAS* mutations, n (%) | 5 (13.9) | 3 (2.1) | **0.008** |
| *TET2* mutations, n (%) | 3 (8.3) | 7 (4.8) | 0.417 |
| *ASXL1* mutations, n (%) | 8 (22.2) | 9 (6.2) | **0.007** |
| *RUNX1* mutations*, n (%)* | 6 (16.7) | 4 (2.7) | **0.005** |
| *SF3B1* mutations, n (%) | 1 (2.8) | 3 (2.1) | 1 |
| *SRSF2* mutations, n (%) | 3 (8.3) | 5 (3.4) | 0.194 |
| Disease modifying therapy for t-MN* | | | |
| No DMT Intensive chemotherapy HMA based chemotherapy Venetoclax based therapy | 6 (16.7) 9 (25.0) 13 (36.1) 5 (13.9) | 28 (19.2) 14 (9.6) 65 (44.5) 33 (22.6) | 0.089 |
| Allogeneic SCT, n (%) | 8 (22.2) | 23 (15.8) | 0.335 |
| Interval between primary to t-MN (months), median (IQR) | 97.1 (60.7, 167.4) | 87.1 (46.5, 154.1) | 0.682 |
| Clinical features at primary disease | | | |
| Age at primary disease, median (IQR) | 52.0 (40.5, 62.0) | 59.0 (51.0, 66.0) | **0.01** |
| Hematology malignancy, n (%)  Solid cancer, n (%)  AID, n (%)  Hem and solid cancer, n (%)  Two independent solid cancers, n (%)  Solid cancer and AID, n (%) | 20 (55.6)  11 (30.6)  3 (8.3)  1 (2.8)  1 (2.8)  0 (0.0) | 84 (57.5)  37 (25.3)  9 (6.2)  7 (4.8)  7 (4.8)  2 (1.4) | 0.907 |
| Chemotherapy for primary cancer/disease | 15 (41.7) | 77 (52.7) | 0.267 |
| Radiation for primary cancer, n (%) | 4 (11.1) | 11 (7.5) | 0.501 |
| Auto SCT for primary cancer, n (%) | 11 (30.6) | 36 (24.7) | 0.525 |
| Immunosuppression, n (%) | 5 (13.9) | 14 (9.6) | 0.541 |

Five *TP53*^mut^ patients had VAF <10% and three VAF NA

*First line of therapy only

**Table S8: Distribution of *TP53* mutated patients according to bone marrow (BM) blast percentage**

| Variables | Bone marrow blast % at diagnosis | | | | *P*-value |
| --- | --- | --- | --- | --- | --- |
|  | <5%  (*n* = 71) | 5-9%  (*n* = 26) | 10-19%  (*n* = 24) | ≥ 20%  (*n* = 49) |  |
| Clinical features at primary disease | | | | | |
| Age at primary disease,  median (IQR) | 60.0 (54.0, 65.0) | 56.0 (48.0, 65.0) | 65.0 (55.5, 69.5) | 53.0 (47.2, 65.7) | 0.059 |
| Hematology malignancy, n (%)  Solid cancer, n (%)  AID, n (%)  Hem and solid cancer, n (%)  Two independent solid cancers, n (%)  Solid cancer and AID, n (%)  Other, n (%) | 43 (60.6)  13 (18.3)  6 (8.5)  6 (8.5)  3 (4.2)  0 (0.0)  0 (0.0) | 12 (46.2)  11 (42.3)  1 (3.8)  0 (0.0)  1 (3.8)  0 (0.0)  1 (3.8) | 12 (50.0)  7 (29.2)  1 (4.2)  2 (8.3)  1 (4.2)  1 (4.2)  0 (0.0) | 25 (51.0)  17 (34.7)  3 (6.1)  0 (0.0)  3 (6.1)  1 (2.0)  0 (0.0) | 0.537 |
| Chemotherapy for primary cancer/disease (%) | 43 (60.6) | 9 (34.6) | 14 (58.3) | 17 (34.7) | **0.013** |
| Radiation for primary  cancer (%) | 5 (7.0) | 4 (15.4) | 0 (0.0) | 7 (14.3) | 0.121 |
| Auto SCT for primary  cancer (%) | 26 (36.6) | 3 (11.5) | 4 (16.7) | 8 (16.3) | **0.018** |
| Immunosuppression (%) | 6 (8.5) | 3 (11.5) | 3 (12.5) | 5 (10.2) | 0.886 |
| Interval between primary to t-MN (months), median (IQR) | 101.5  (44.7, 148.9) | 87.2  (63.9, 154.1) | 79.3  (50.0, 127.5) | 108.0  (40.7, 157.9) | 0.752 |
| Clinical features at t-MN diagnosis | | | | | |
| Age at t-MN, median (IQR) | 67.3 (62.4, 74.3) | 65.9 (59.5, 72.6) | 71.8 (67.0, 75.1) | 68.0 (61.0, 74.6) | 0.201 |
| Female/male | 26/45 | 11/15 | 10/14 | 19/30 | 0.937 |
| Hb g/dL, median (IQR) | 9.10 (8.12, 11.00) | 8.90 (8.00, 9.57) | 9.30 (8.28, 10.05) | 8.30 (7.50, 9.57) | 0.128 |
| WBC x 10^9^/L, median (IQR) | 3.20 (2.28, 4.27) | 2.69 (1.85, 3.85) | 2.00 (1.20, 4.12) | 2.60 (1.30, 4.60) | 0.09 |
| ANC x 10^9^/L, median (IQR) | 1.22 (0.68, 1.89) | 1.07 (0.50, 2.30) | 0.69 (0.51, 1.22) | 0.62 (0.22, 1.43) | **0.032** |
| Platelets x 10^9^/L, median (IQR) | 68.5 (33.2, 98.5) | 63.0 (40.0, 96.7) | 34.0 (21.7, 74.7) | 48.0 (27.0, 95.0) | 0.064 |
| Cytogenetic changes | | | | | |
| Number of cases with cytogenetic aberrancy, n (%) | 69 (97.2) | 25 (96.2) | 24 (100.0) | 47 (95.9) | 1 |
| Complex karyotype, n (%) | 61 (85.9) | 23 (88.5) | 24 (100.0) | 38 (77.6) | 0.055 |
| Monosomal karyotype, n (%) | 56 (78.9) | 21 (80.8) | 19 (79.2) | 41 (83.7) | 0.917 |
| Marker chromosome, n (%) | 31 (43.7) | 14 (53.8) | 14 (60.9) | 31 (63.3) | 0.166 |
| Ring chromosome, n (%) | 16 (22.5) | 7 (26.9) | 5 (21.7) | 12 (24.5) | 0.967 |
| Del 5q/ Monosomy 5, n (%) | 41 (57.7) | 17 (65.4) | 16 (66.7) | 19 (38.8) | 0.051 |
| Del 7q/ Monosomy 7, n (%) | 38 (53.5) | 16 (61.5) | 10 (41.7) | 19 (38.8) | 0.193 |
| Trisomy 8, n (%) | 10 (20.4) | 3 (16.7) | 4 (23.5) | 7 (20.6) | 0.449 |
| Abnormal Chrom. 9, n (%) | 13 (18.3) | 1 (3.8) | 5 (20.8) | 16 (32.7) | **0.024** |
| Abnormal Chrom. 11, n (%) | 11 (15.5) | 7 (26.9) | 11 (45.8) | 16 (32.7) | **0.017** |
| Abnormal Chrom. 12, n (%) | 23 (32.4) | 10 (38.5) | 9 (37.5) | 12 (24.5) | 0.526 |
| Abnormal Chrom. 13, n (%) | 19 (26.8) | 6 (23.1) | 9 (37.5) | 20 (40.8) | 0.275 |
| Abnormal Chrom. 16, n (%) | 10 (14.1) | 3 (11.5) | 7 (29.2) | 21 (42.9) | **0.001** |
| Abnormal Chrom. 3, n (%) | 16 (22.5) | 6 (23.1) | 7 (29.2) | 19 (38.8) | 0.258 |
| Abnormal Chrom. 18, n (%) | 16 (22.5) | 8 (30.8) | 12 (50.0) | 19 (38.8) | 0.055 |
| Abnormal Chrom. 19, n (%) | 12 (16.9) | 4 (15.4) | 6 (25.0) | 18 (36.7) | 0.067 |
| Abnormal Chrom. 20, n (%) | 15 (21.1) | 6 (23.1) | 8 (33.3) | 13 (26.5) | 0.644 |
| Abnormal Chrom. 21, n (%) | 14 (19.4) | 9 (34.6) | 9 (37.5) | 25 (51.0) | **0.004** |
| Somatic mutations on NGS | | | | | |
| *TP53* mutation *±* 17p13 loss Single-hit (%) Multi-hit (%) VAF, median (IQR) | 17 (23.9) 54 (76.1) 37.7 (19.6, 46.5) | 3 (11.5) 23 (88.5) 37.0 (31.4, 48.3) | 3 (12.5) 21 (87.5) 34.0 (24.2, 42.0) | 10 (20.4) 39 (79.6) 40.0 (25.0, 59.3) | 0.508  0.593 |
| Co-mutations excluding *TP53*  ≥2 mutations, n (%) 1 mutation, n (%) No mutation, n (%) | 11 (15.5) 16 (22.5) 44 (62.0) | 3 (11.5) 5 (19.2) 18 (69.2) | 2 (8.3) 8 (33.3) 14 (58.3) | 8 (16.3) 13 (26.5) 28 (57.1) | 0.881 |
| *DNMT3A* mutations, n (%) | 10 (14.1) | 2 (7.7) | 2 (8.3) | 6 (12.2) | 0.861 |
| *RAS* mutations, n (%) | 1 (1.4) | 0 (0.0) | 0 (0.0) | 3 (6.1) | 0.328 |
| *TET2* mutations, n (%) | 5 (7.0) | 0 (0.0) | 2 (8.3) | 4 (8.2) | 0.553 |
| *ASXL1* mutations, n (%) | 4 (5.6) | 1 (3.8) | 2 (8.3) | 4 (8.2) | 0.835 |
| *RUNX1* mutations, n (%) | 4 (5.6) | 1 (3.8) | 0 (0.0) | 3 (6.1) | 0.838 |
| *SF3B1* mutations, n (%) | 3 (4.2) | 1 (3.8) | 0 (0.0) | 1 (2.0) | 0.864 |
| *SRSF2* mutations, n (%) | 2 (2.8) | 1 (3.8) | 1 (4.2) | 2 (4.1) | 1 |
| Disease modifying therapy for t-MN* | | | | | |
| No DMT, n (%) Intensive chemotherapy, n (%) HMA based chemotherapy, n (%) Venetoclax based therapy, n (%) | 20 (28.2) 3 (4.2) 38 (53.5) 5 (7.0) | 5 (19.2) 3 (11.5) 17 (65.4) 1 (3.8) | 2 (8.3) 1 (4.2) 11 (45.8) 10 (41.7) | 5 (10.2) 13 (26.5) 7 (14.3) 23 (46.9) | 0.438 |
| Allogeneic SCT, n (%) | 13 (18.3) | 6 (23.1) | 0 (0.0) | 5 (10.2) | **0.041** |

Abbreviations: AID – Autoimmune disease; t-MN – therapy related myeloid neoplasm; Hb – Hemoglobin; WBC – white blood cell; ANC – absolute neutrophil count; Chrom. – Chromosome; VAF – Variant Allele Frequency; DMT – disease-modifying therapy; HMA – hypomethylating agents; SCT – Stem Cell Transplant.

*As first line of therapy

**Table S9: Cox regression univariate analysis of variables predicting OS in *TP53*^mut^ t-MN**

| Variables | HR (95% CI) | *P-*value |
| --- | --- | --- |
| Clinical features at t-MN diagnosis |  |  |
| Age at t-MN diagnosis | 1.02 (0.99-1.03) | 0.105 |
| Gender (Reference - Female) | 1.26 (0.89-1.79) | 0.19 |
| Hb g/dL at t-MN diagnosis | 0.83 (0.76-0.91) | **<0.0001** |
| Platelets x10^9^/L at t-MN diagnosis | 0.996 (0.992-0.999) | **0.013** |
| WBC x10^9^/L at t-MN diagnosis | 1 (0.99-1.02) | 0.531 |
| ANC x10^9^/L at t-MN diagnosis | 1.05 (0.96-1.15) | 0.276 |
| BM blasts (Reference - <5%) |  |  |
| BM blasts 5-9% | 1.08 (0.652-1.8) | 0.76 |
| BM blasts 10-19% | 1.73 (1.03-2.9) | **0.036** |
| BM blasts ≥ 20% | 2.01 (1.33-3.03) | **0.0009** |
| Cytogenetics |  |  |
| Complex karyotype (Reference - No) | 0.78 (0.48-1.27) | 0.322 |
| Monosomal karyotype (Reference - No) | 1.35 (0.859-2.11) | 0.194 |
| Marker chromosome (Reference - No) | 1.16 (0.82-1.64) | 0.397 |
| Abnormal Chrom. 12 (Reference - No) | 0.73 (0.50-1.07) | **0.104** |
| Ring chromosome (Reference - No) | 1.13 (0.76-1.68) | 0.533 |
| Abnormal Chrom. 5 (Reference - No) | 0.968 (0.69-1.36) | 0.85 |
| Abnormal Chrom. 7 (Reference - No) | 1 (0.71-1.4) | 0.998 |
| Abnormal Chrom. 17 (Reference - No) | 1.24 (0.87-1.77) | 0.231 |
| Abnormal Chrom. 11 (Reference - No) | 1.71 (1.18-2.48) | **0.004** |
| Abnormal Chrom. 19 (Reference - No) | 1.74 (1.18-2.57) | **0.005** |
| Monosomy 17 | 1.97 (1.32-2.96) | **0.0009** |
| Monosomy 21 | 1.88 (1.18-2.98) | **0.007** |
| *RAS* mutations, n (%) | 6.97 (2.48-19.6) | **0.0002** |
| t (11q) | 3.75 (1.79-7.85) | **0.0004** |
| Disease phenotype: t-AML  (Reference - t-MDS) | 1.79 (1.24-2.59) | **0.001** |
| Disease modifying therapy for t-MN (Reference - Supportive therapy) |  |  |
| HMA based chemotherapy | 0.396 (0.255-0.616) | **<0.0001** |
| Intensive chemotherapy | 0.63 (0.35-1.13) | 0.119 |
| Venetoclax based therapy | 0.53 (0.32-0.89) | **0.016** |
| Allogeneic SCT for t-MN | 0.282 (0.16-0.49) | **<0.0001** |

**Supplementary Figures**

**
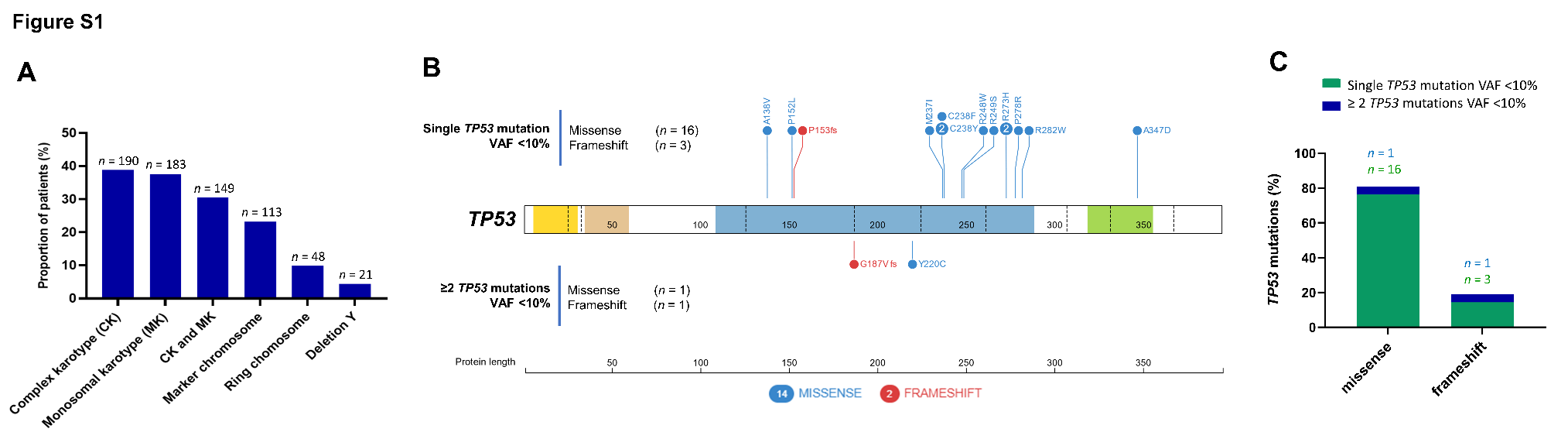
**

**Figure S1. Landscape of chromosomal aberrations in t-MN and distribution of *TP53*^mut^ <10% VAF.** (A) Most common structural chromosomal aberration in t-MN; (B) Distribution of *TP53*^mut^ with VAF <10% along the gene. Mutations from patients with single mutations are shown at the top and those from patients with ≥2 mutations are shown at the bottom. Missense mutations are shown as blue circles and frameshift deletions or insertions as red circles. Functional protein domains are indicated in yellow (transactivation motif), brown (transactivation domain 2), blue (DNA binding domain) and green (tetramerization motif); (C) Summary of *TP53*^mut^ VAF <10% separated by mutation type.

**
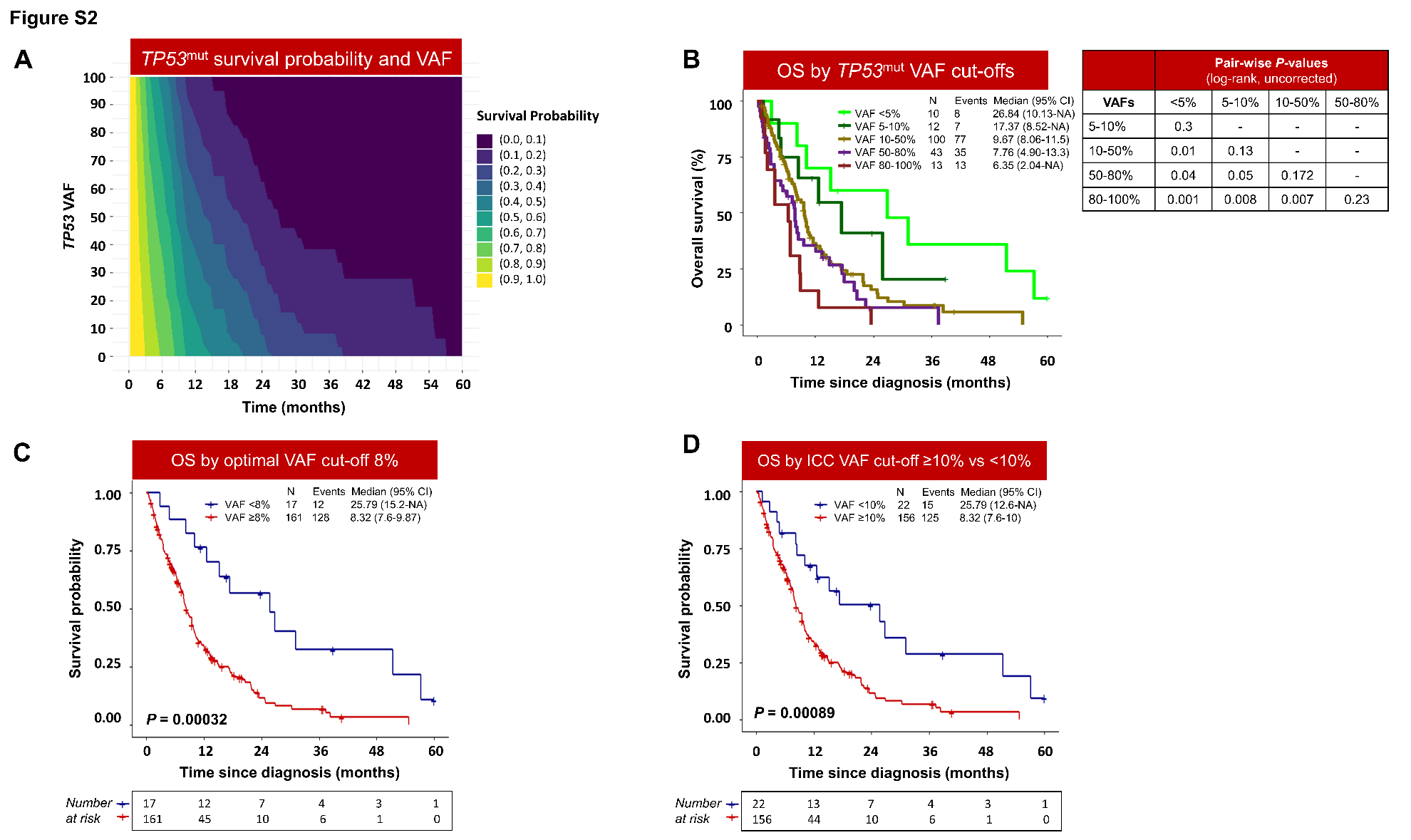
**

**Figure S2. Optimal *TP53*^mut^ VAF threshold associated with poor prognosis in t-MN.** (A) Survival contour plot showing probability of survival according to *TP53*^mut^ VAF, plot was generated to visualize the causal effect of *TP53* VAF on a time-to-event OS outcome; (B) OS according to *TP53*^mut^ VAF cut-offs; (C) The *TP53*^mut^ VAF of 8% was identified as optimal threshold based on maximally selected rank statistics; (D) OS according to ICC cut-off of *TP53*^mut^ VAF ≥10%.

**
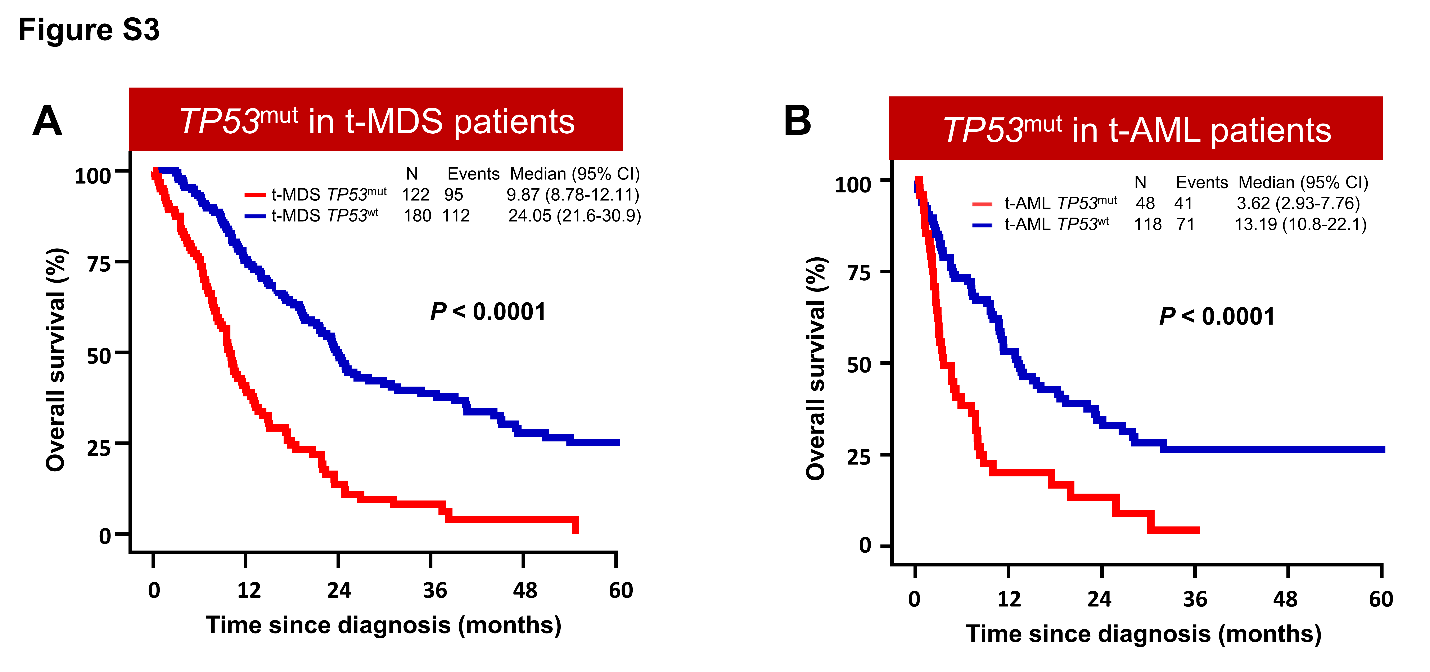
**

**Figure S3.** ***TP53*^mut^ is associated with significantly poor survival across t-MDS and t-AML.** Kaplan-Meier probability estimates for the overall survival in *TP53*^wt^ vs *TP53*^mut^ across: (A) t-MDS and (B) t-AML. The median, 95% confidence interval (CI) and *P* values observed for survival curves are indicated.

**
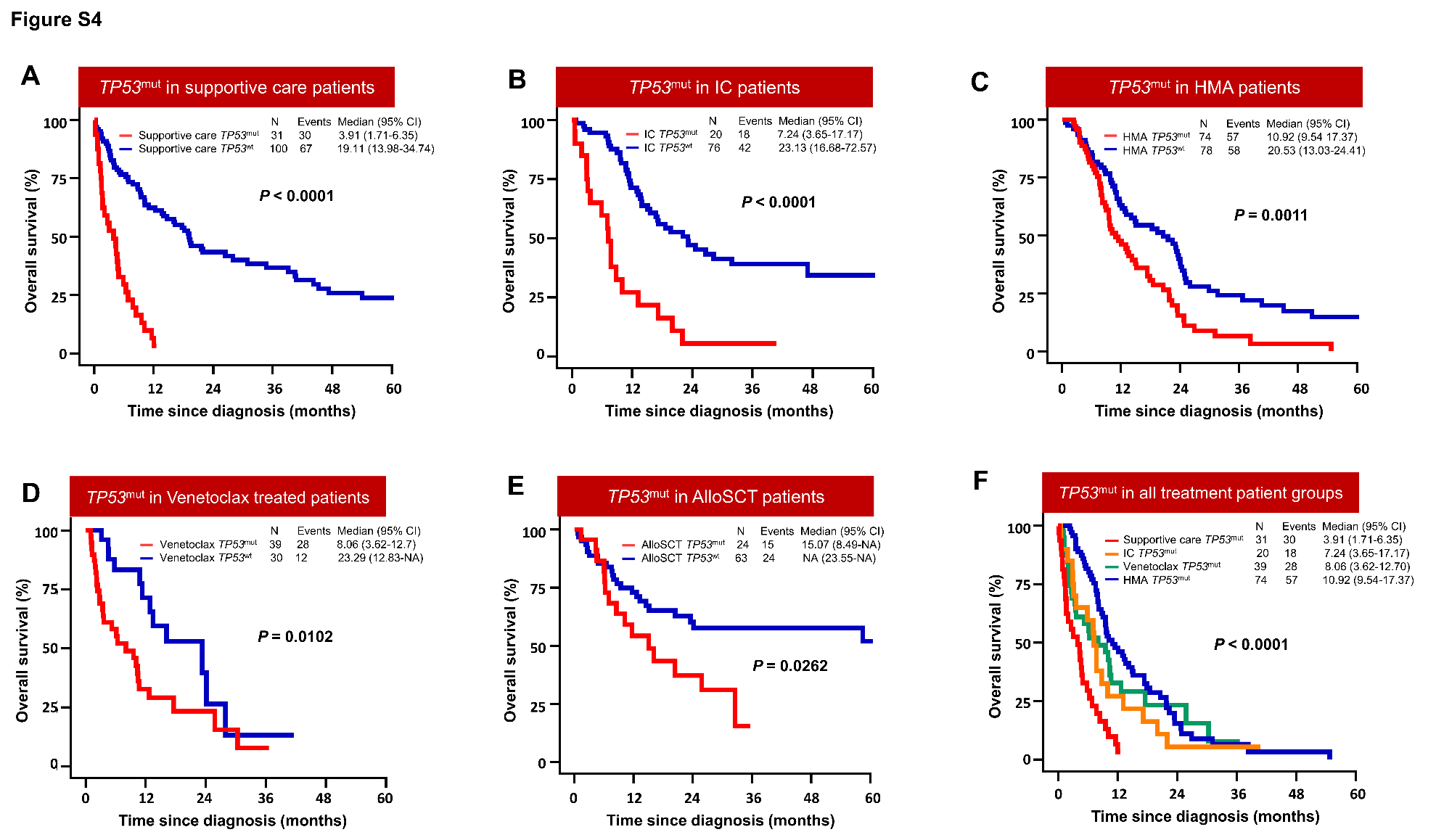
**

**Figure S4. *TP53*^mut^ is associated with significantly poor survival across treatment types.** Kaplan-Meier probability estimates for the OS in *TP53*^wt^ vs *TP53*^mut^ across first line therapy: (A) Supportive care, (B) Intensive chemotherapy (IC); (C) Hypomethylating agents (HMA); (D) Venetoclax based therapies; (E) Allogeneic SCT (AlloSCT); (F) Comparison of OS of *TP53*^mut^ according to first line disease modifying therapies.

**
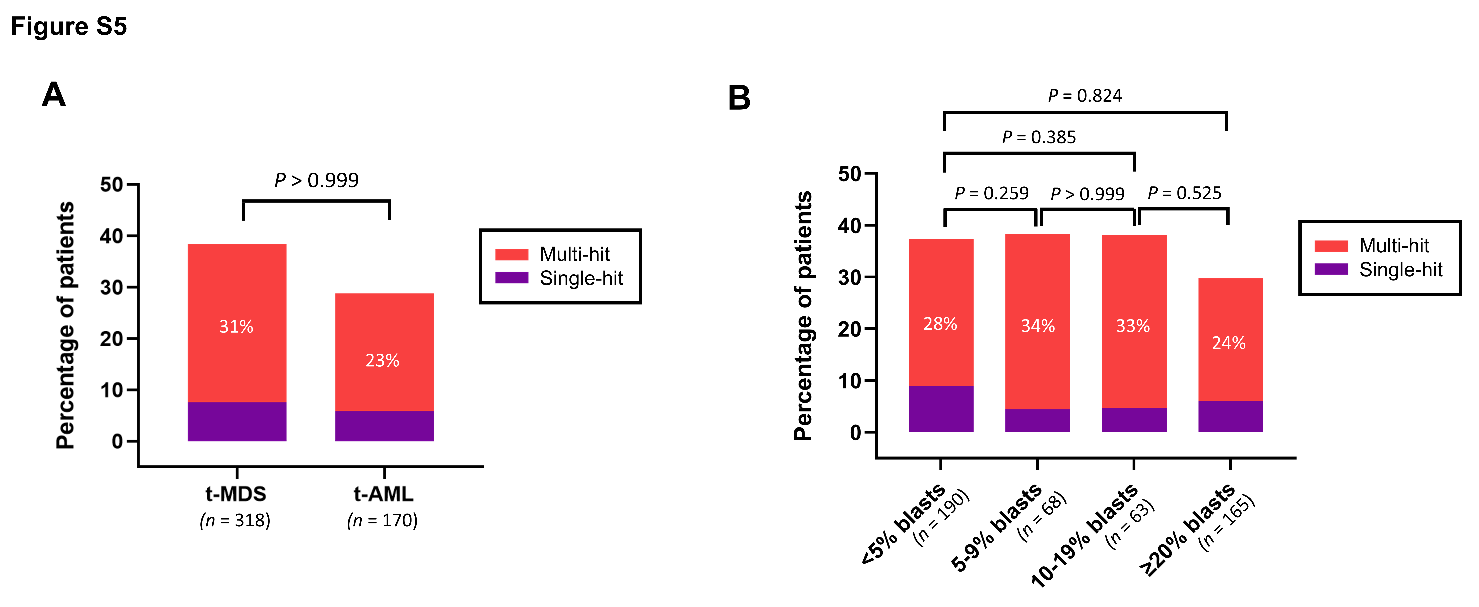
**

**Figure S5. Distribution of *TP53*^mut^ allelic status according to t-MN phenotype and BM blast categories.** Single- and multi-hit status was similar in (A) t-MDS and t-AML; and (B) in BM blast categories.

**
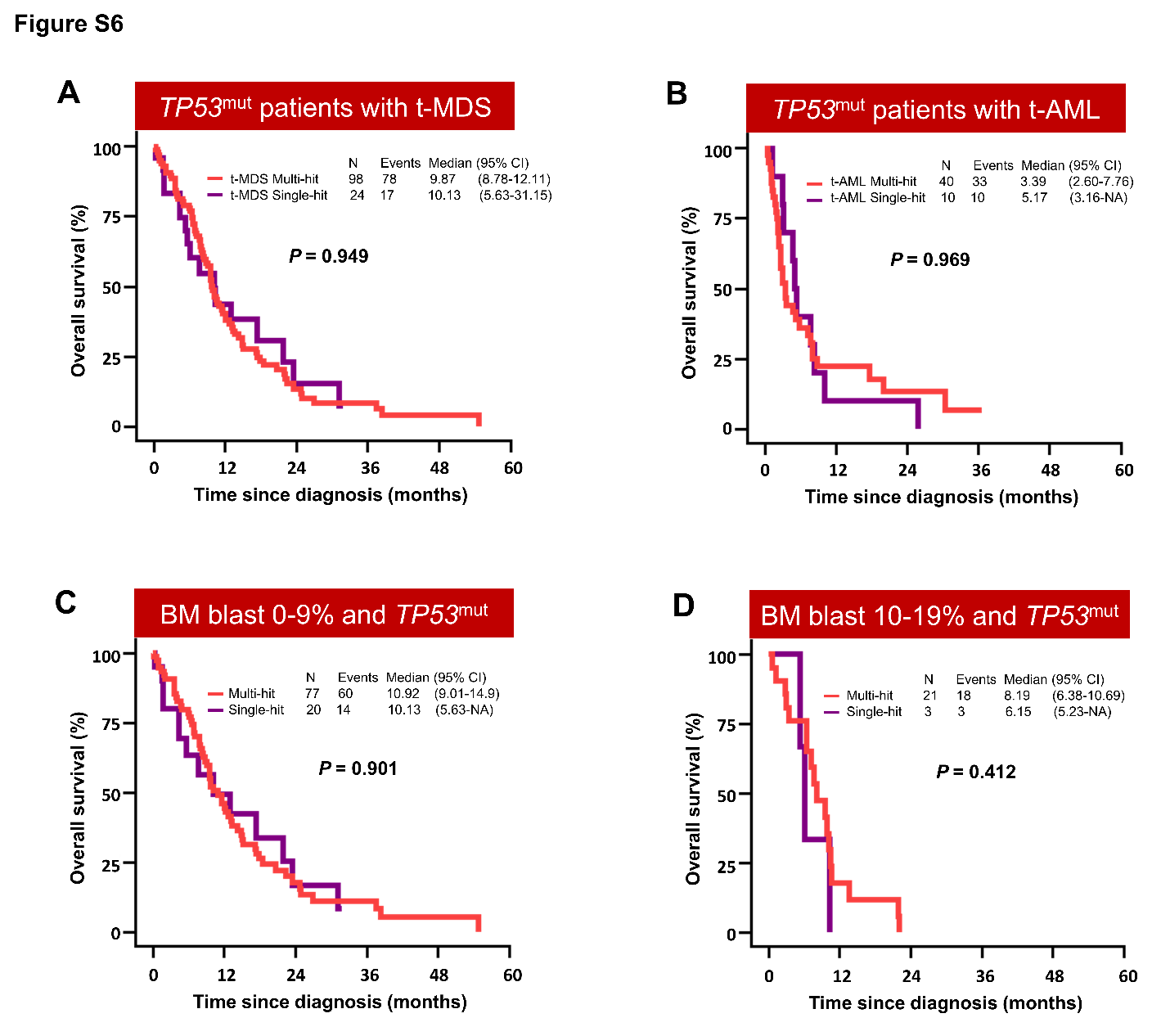
**

**Figure S6. Overall survival of single- or multi-hit *TP53*^mut^ according to t-MN phenotype and BM blast categories of t-MDS proposed by International Consensus Classification.** OS of single- vs multi-hit *TP53*^mut^ is equally poor in (A) t-MDS; (B) t-AML; (C) BM blasts 0-9%; and (D) BM blasts 10-19% categories.

**
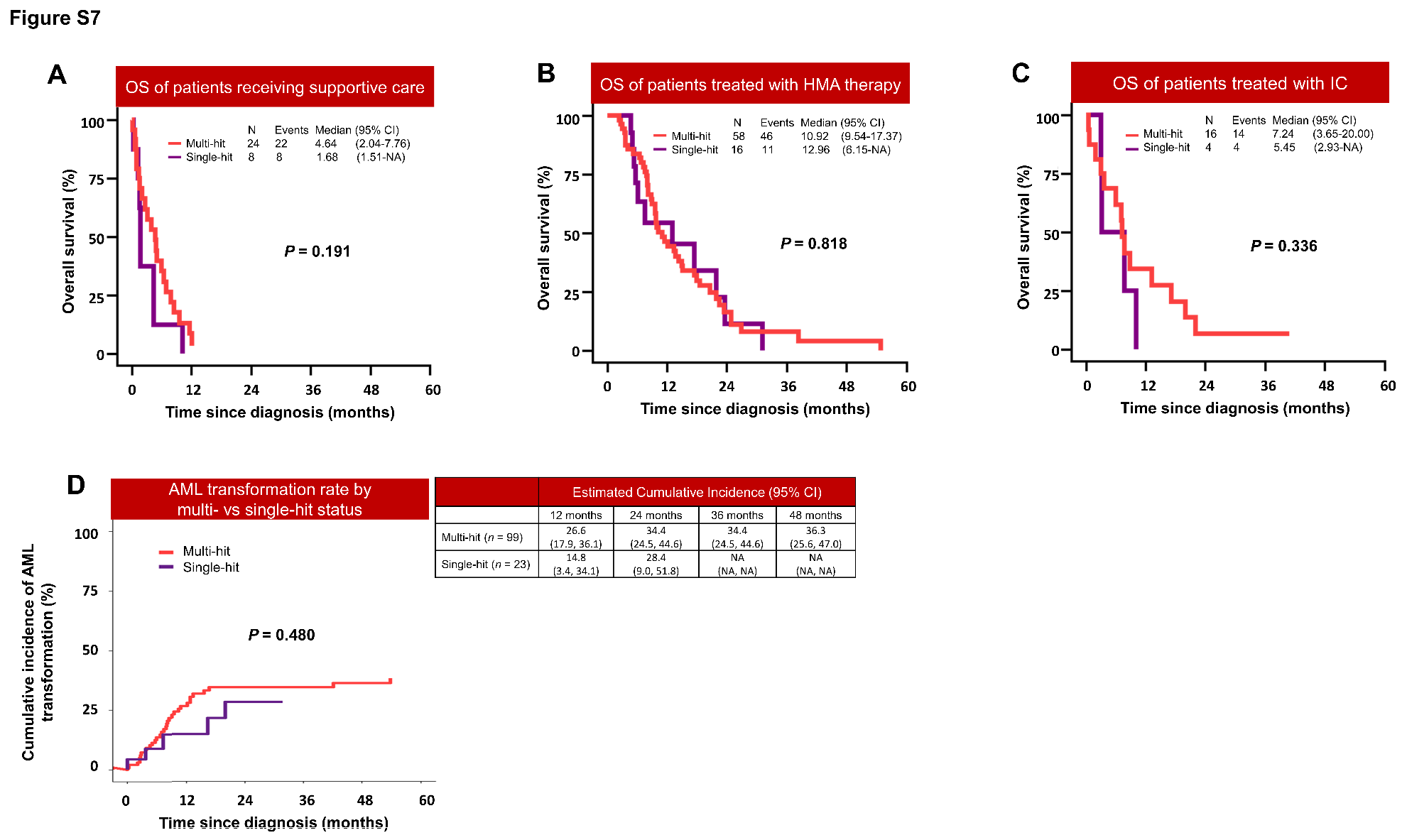
**

**Figure S7. Overall survival of single- or multi-hit *TP53*^mut^ across of first-line treatment.** OS of single- vs multi-hit *TP53*^mut^ is equally poor in t-MN treated with (A) Supportive care; (B) HMA therapy; (C) Intensive chemotherapy (IC); (D) Cumulative incidence of AML transformation of multi-hit vs single-hit patients.

**
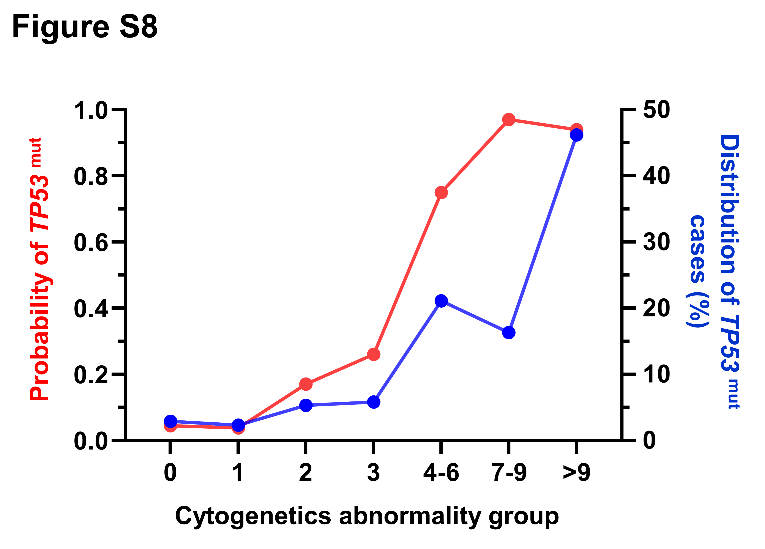
**

**Figure S8. Probabilities and distribution of *TP53*^mut^ according to number of structural cytogenetic abnormalities.** *TP53*^mut^ (80%) were segregated in t-MN with $\geq$4 cytogenetic abnormalities.

**
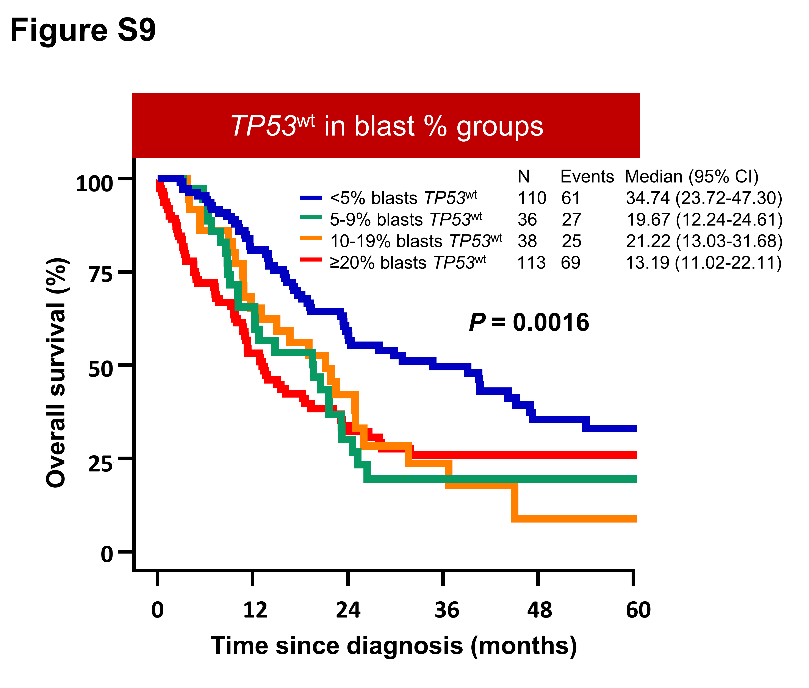
**

**Figure S9.** **Kaplan-Meier probability estimates for the OS of *TP53* wild-type (*TP53*^wt^) according to BM blast %.**

**References**

1. Arber, D. A., Orazi, A., Hasserjian, R., Thiele, J., Borowitz, M. J., Le Beau, M. M. *et al.* The 2016 revision to the World Health Organization classification of myeloid neoplasms and acute leukemia. *Blood* **127**, 2391-2405.

2. Hiwase, D. K., Hahn, C. N., Tran, E. N. H., Chhetri, R., Baranwal, A., Al-Kali, A. *et al.* TP53 mutation in therapy-related myeloid neoplasm defines a distinct molecular subtype. *Blood* 10.1182/blood.2022018236.

3. Chun, K., Hagemeijer, A., Iqbal, A. & Slovak, M. L. Implementation of standardized international karyotype scoring practices is needed to provide uniform and systematic evaluation for patients with myelodysplastic syndrome using IPSS criteria: An International Working Group on MDS Cytogenetics Study. *Leuk Res* **34**, 160-165.

4. Haase, D., Stevenson, K. E., Neuberg, D., Maciejewski, J. P., Nazha, A., Sekeres, M. A. *et al.* TP53 mutation status divides myelodysplastic syndromes with complex karyotypes into distinct prognostic subgroups. *Leukemia* **33**, 1747-1758.

5. Mrozek, K. Cytogenetic, molecular genetic, and clinical characteristics of acute myeloid leukemia with a complex karyotype. *Semin Oncol* **35**, 365-377.

6. Mrozek, K., Eisfeld, A. K., Kohlschmidt, J., Carroll, A. J., Walker, C. J., Nicolet, D. *et al.* Complex karyotype in de novo acute myeloid leukemia: typical and atypical subtypes differ molecularly and clinically. *Leukemia* **33**, 1620-1634.

7. Döhner, H., Wei, A. H., Appelbaum, F. R., Craddock, C., DiNardo, C. D., Dombret, H. *et al.* Diagnosis and Management of AML in Adults: 2022 ELN Recommendations from an International Expert Panel. *Blood* 10.1182/blood.2022016867, blood.2022016867.

8. McGowan-Jordan, J., Simons, A. & Schmid, M. *ISCN 2016: An International System for Human Cytogenomic Nomenclature (2016)*. (Karger, 2016).

9. Branford, S., Wang, P., Yeung, D. T., Thomson, D., Purins, A., Wadham, C. *et al.* Integrative genomic analysis reveals cancer-associated mutations at diagnosis of CML in patients with high-risk disease. *Blood* **132**, 948-961.

10. Mehta, N., He, R. & Viswanatha, D. S. Internal Standardization of the Interpretation and Reporting of Sequence Variants in Hematologic Neoplasms. *Mol Diagn Ther* **25**, 517-526.

11. Singhal, D., Wee, L. Y. A., Kutyna, M. M., Chhetri, R., Geoghegan, J., Schreiber, A. W. *et al.* The mutational burden of therapy-related myeloid neoplasms is similar to primary myelodysplastic syndrome but has a distinctive distribution. *Leukemia* **33**, 2842-2853.

12. Li, M. M., Datto, M., Duncavage, E. J., Kulkarni, S., Lindeman, N. I., Roy, S. *et al.* Standards and Guidelines for the Interpretation and Reporting of Sequence Variants in Cancer: A Joint Consensus Recommendation of the Association for Molecular Pathology, American Society of Clinical Oncology, and College of American Pathologists. *J Mol Diagn* **19**, 4-23.

13. Bernard, E., Nannya, Y., Hasserjian, R. P., Devlin, S. M., Tuechler, H., Medina-Martinez, J. S. *et al.* Implications of TP53 allelic state for genome stability, clinical presentation and outcomes in myelodysplastic syndromes. *Nature Medicine* **26**, 1549-1556.

14. Lindsley, R. C., Saber, W., Mar, B. G., Redd, R., Wang, T., Haagenson, M. D. *et al.* Prognostic Mutations in Myelodysplastic Syndrome after Stem-Cell Transplantation. *New England Journal of Medicine* **376**, 536-547.

15. Arber, D. A., Orazi, A., Hasserjian, R. P., Borowitz, M. J., Calvo, K. R., Kvasnicka, H. M. *et al.* International Consensus Classification of Myeloid Neoplasms and Acute Leukemia: Integrating Morphological, Clinical, and Genomic Data. *Blood* 10.1182/blood.2022015850.

16. Lausen, B. & Schumacher, M. Maximally Selected Rank Statistics. *Biometrics* **48**, 73-85.
